# Denatonium as a Bitter Taste Receptor Agonist Modifies Transcriptomic Profile and Functions of Acute Myeloid Leukemia Cells

**DOI:** 10.3389/fonc.2020.01225

**Published:** 2020-07-24

**Authors:** Valentina Salvestrini, Marilena Ciciarello, Valentina Pensato, Giorgia Simonetti, Maria Antonella Laginestra, Samantha Bruno, Martina Pazzaglia, Elena De Marchi, Dorian Forte, Stefania Orecchioni, Giovanni Martinelli, Francesco Bertolini, Simon Méndez-Ferrer, Elena Adinolfi, Francesco Di Virgilio, Michele Cavo, Antonio Curti

**Affiliations:** ^1^Department of Experimental, Diagnostic and Specialty Medicine, Policlinico S. Orsola-Malpighi University Hospital, University of Bologna, Bologna, Italy; ^2^Istituto Scientifico Romagnolo per lo Studio e la Cura dei Tumori IRCCS, Meldola, Italy; ^3^Laboratory of Experimental Oncology, IRCCS Istituto Ortopedico Rizzoli, Bologna, Italy; ^4^Department of Morphology, Surgery and Experimental Medicine, Section of Pathology, Oncology and Experimental Biology, University of Ferrara, Ferrara, Italy; ^5^Laboratory of Hematology-Oncology, IRCCS European Institute of Oncology, Milan, Italy; ^6^Department of Haematology, Wellcome Trust-Medical Research Council Cambridge Stem Cell Institute, University of Cambridge, Cambridge, United Kingdom; ^7^Centro Nacional de Investigaciones Cardiovasculares, Madrid, Spain; ^8^Department of Oncology and Hematology, Institute of Hematology “L. and A. Seràgnoli”, University-Hospital S.Orsola-Malpighi, Bologna, Italy

**Keywords:** acute myeloid leukemia, bitter taste receptors, denatonium benzoate, bone marrow microenvironment, bitter compounds

## Abstract

The contribution of cell-extrinsic factors in Acute Myeloid Leukemia (AML) generation and persistence has gained interest. Bitter taste receptors (TAS2Rs) are G protein-coupled receptors known for their primary role as a central warning signal to induce aversion toward noxious or harmful substances. Nevertheless, the increasing amount of evidence about their extra-oral localization has suggested a wider function in sensing microenvironment, also in cancer settings. In this study, we found that AML cells express functional TAS2Rs. We also highlighted a significant association between the modulation of some TAS2Rs and the poor-prognosis AML groups, i.e., *TP53*- and *TET2*-mutated, supporting a potential role of TAS2Rs in AML cell biology. Gene expression profile analysis showed that TAS2R activation with the prototypical agonist, denatonium benzoate, significantly modulated a number of genes involved in relevant AML cellular processes. Functional assay substantiated molecular data and indicated that denatonium reduced AML cell proliferation by inducing cell cycle arrest in G0/G1 phase or induced apoptosis via caspase cascade activation. Moreover, denatonium exposure impaired AML cell motility and migratory capacity, and inhibited cellular respiration by decreasing glucose uptake and oxidative phosphorylation. In conclusion, our results in AML cells expand the observation of cancer TAS2R expression to the setting of hematological neoplasms and shed light on a role of TAS2Rs in the extrinsic regulation of leukemia cell functions.

## Introduction

Acute Myeloid Leukemia (AML) is a clonal disease developing from a rare population of leukemic stem cells. Alongside the identification of disease-specific alleles harbored by AML clones, the contribution that cell-extrinsic factors have in AML generation and persistence by influencing AML cell genomic landscape and therapy-resistance is gaining increasing interest (1, 2). In the cross-talk between AML cells and their microenvironment, several membrane receptors crucially sense and respond to external changes by triggering those intracellular signals, which in turn activate specific pathways. Among these receptors, the largest group belongs to the family of G protein-coupled receptors (GPCRs).

In humans, bitter taste receptors (TAS2Rs) comprise 25 distinct members of the GPCR family (3). Initially described in the oral cavity, TAS2Rs are well-known to mediate the perception of bitter taste and are associated with a self-defense system against the ingestion of dangerous and toxic substances (4). Intriguingly, recent studies have shown that TAS2Rs are also expressed in many extra-oral tissues, such as the respiratory and endocrine system, the gastrointestinal tract, reproductive tissue and the brain (5–9). Although these extra-oral functions are still poorly understood, some evidence suggests that TAS2Rs may represent a receptor system, used by different cell types to sense external stimuli (10–13). Indeed, in the gastrointestinal tract TAS2Rs function by sensing luminal content and hormones (10). Throughout the respiratory epithelium, TAS2Rs are found in solitary chemosensory cells and in the airway smooth muscle, where they respectively mediate protective airway reflex and bronchodilation (11, 12). In the upper respiratory tract, TAS2Rs are directly activated by bacterial products and contribute to immune responses by favoring the production of antimicrobial substances from mucosal cells (13).

Cancer cells, including leukemia cells, are known to adapt and turn ancestral and phylogenetically conserved functional pathways into malignant settings (14). Notably, TAS2R expression has recently been reported in diverse tumor types, including neuroblastoma (15), pancreatic (16), prostate (17), ovarian (17), and breast cancer (18). However, no clear evidence of their function has been provided, although a potential role in cancer biology has been recently suggested. A stimulation of T2R38 in pancreatic cancer upregulates the multi drug resistance protein ABCB1, a major player in the induction of chemoresistance (19). Singh et al. demonstrated a differential expression of TAS2Rs in non-cancerous breast epithelial vs. breast cancer cells, with some TAS2Rs downregulated in breast cancer cells (18). Moreover, it has been reported that TAS2R4 and TAS2R14 are involved in the regulation of proliferation and migration of highly metastatic breast cancer cells (20). In neuroblastoma cells, TAS2R8 and TAS2R10 play an important role in inhibiting the self-renewal potential and the invasion ability of cancer cells (15). On the other hand, several bitter compounds and several agonist for TAS2Rs have has been found to exhibit anticancer or chemotherapy enhancing activities, whereby the exact mechanisms are often unknown (21–27).

In this study, for the first time, we characterized TAS2R expression in AML cells and we investigated the effects of a stimulation with denatonium benzoate (DEN), a widely used bitter taste agonist which has been demonstrate to activate TAS2Rs on various cell types (10, 28–31). Our data provide evidence that bitter compounds can modulate leukemia cell genetic profile and functions.

## Methods

### Cell Isolation and Culture

Primary leukemic cells (AML cells) from 47 patients at diagnosis (blasts > 80%) were obtained from bone marrow (BM) or peripheral blood (PB), upon signed informed consent (Table S1). Mononuclear cells were separated by density gradient centrifugation (Lympholyte, Cedarlane, Burlington, Canada). Human leukemia cell lines OCI-AML3, THP-1 were purchased from DSMZ (Braunschweig, Germany) and used between passages 10 and 25. Knockout (CRISPR-Cas9 targeting TAS2R4, TAS2R10, TAS2R8, TAS2R13, or TAS2R30 genes) THP-1 cells were purchased from Synthego Inc (Redwood City, CA, USA). All cells culture were maintained at the concentration of 5 x 10^5^/ml in RPMI 1640 medium supplemented with 10% FBS (Thermo Fisher Scientific, Waltham, MA, USA) at 37°C, 5% CO_2_, w or w/o increasing doses of denatonium benzoate (dissolved in RPMI 1640 medium), quinine and chloroquine (dissolved in ethanol and water, respectively) (Sigma Aldrich, St. Louis, MO, USA). This research was approved by the Ethics Committee of Policlinico S. Orsola-Malpighi, University Hospital of Bologna (approval code: 94/2016/O/Tess).

### Gene Expression Profiling (GEP)

TAS2R expression was analyzed in 61 AML, 49 from a published dataset (32) and 12 new cases. As validation set, we used also 183 AML samples downloaded from The Cancer Genome Atlas (TCGA) (https://gdc.cancer.gov/about-data/publications/laml_2012) (33). GEP after DEN treatment was performed in 5 newly diagnosed AML samples and THP-1 and OCI-AML3 cell lines. Three independent replicates of each condition were hybridized to Human Clariom S Arrays (Thermo Fisher Scientific) according to the manufacturer's recommendations. Data quality control, normalization (signal space transformation robust multiple-array average), and supervised analysis were carried out by Expression Console and Transcriptome Analysis Console software, respectively (Thermo Fisher Scientific). For AML cells, data were normalized on vehicle-treated cells before comparison. Genes with a 1.5 fold difference and *p* ≤ 0.05 were considered for enrichment analyses. Downstream analyses were performed as reported in (32, 34), and with Thomson Reuter's MetaCore software suite (Clarivate Analytics, Philadelphia, PA, USA). Gene expression data of denatonium-treated cells will be publicly available on the GEO database under the accession number GSE149548.

### qRT-PCR

MNCs were isolated from BM aspirates of AML samples at diagnosis. Fresh isolated cells were lysed in RLTplus buffer (Qiagen, Hilden) and stored at −80°C for the following steps. Stored RLT lysed were defrosted all at once and processed for RNA extraction as described above.

AML cell lines, cultured as described before, were seeded at 5 x 10^5^/ml and the day after were lysed in RLTplus buffer, stored at −80°C and processed as described for AML primary samples.

Total RNA was isolated using a Rneasy Micro kit (Qiagen) according to the manufacturer's instructions and quantified by Nanodrop ND-1000 spectrophotometer (Thermo Fisher Scientific). RNA samples were treated with DNase (Thermo Fisher Scientific) and reverse transcribed (35). The qRT-PCR reactions were performed using a 96-well Optical Reaction Plate and an ABI-PRISM 7900 Sequence Detection System (Thermo Fisher Scientific) (35). The threshold cycle (C_t_) values for target genes and endogenous reference gene (Table S2) were determined automatically. Relative quantification was calculated using the ΔCt comparative method (34). cDNA from Universal RNA (Agilent genomics, Santa Clara, CA, USA) was used as reference sample. All reactions were performed in duplicate.

### Cytosolic Ca^2+^ Concentration Measurements

Cytosolic free Ca^2+^ concentrations were measured in a thermostat-controlled (37°C) and magnetically-stirred Cary Eclipse Fluorescence Spectrophotometer (Agilent Technologies) with the fluorescent indicator fura-2/AM (36). Briefly, 2 × 10^6^ cells were loaded with 2 μM fura-2/AM for 20 m in the presence of 1 mM CaCl_2_ and 250 μM sulfinpyrazone in the following saline solution: 125 mM NaCl, 5 mM KCl, 1 mM MgSO_4_, 1 mM NaH_2_PO_4_, 20 mM HEPES, 5.5 mM glucose, 5 mM NaHCO_3_, pH 7.4. Subsequently, cells were rinsed, and resuspended at a final concentration of 1 x 10^6^/ml in the same buffer supplemented, whenever required, with 1 mM CaCl_2_, or 500 μM EDTA or 10 μM BAPTA-AM. In the latter case, to ensure for BAPTA-AM entry inside the intracellular compartments and complete chelation of stores' calcium cells were also pre-incubated with BAPTA-AM at 37°C for 30 min before proceeding to fluorimetric measurements. Cells were stimulated with 10 mM DEN or 75 μM quinine, following signal stabilization. Excitation ratio and emission wavelengths were 340/380 and 505 nm, respectively.

### Western Blot Analysis

AML cells were lysed at 4°C for 30 min in Cell Lysis Buffer (Cell Signaling Technology, Danvers, MA, USA), with 1 mM Phenylmethanesulfonyl fluoride (Sigma Aldrich). Protein extracts (30 μg) were separated using a 10–12% Mini-PROTEAN® TGX Stain-Free Precast Gels (Bio-Rad, Hercules, CA, USA), transferred on membranes and incubated with the indicated antibodies (Table S3). Reactive proteins were revealed using ECL Select (GE Healthcare, Chicago, IL, USA). Precision Plus Protein Kaleidoscope (Bio-Rad) was used as a protein molecular weight standard.

### Viability and Proliferation Assay

5 x 10^5^ cells/100 μl culture medium were seeded into a 96-well microplate and treated as indicated. After culture, CellTiter 96 AQueous One Solution reagent (Promega, Madison, WI, USA) was added to each well and the microplate was incubated for 4 h in standard conditions, according to protocol's instruction. The optical density value was measured by an ELISA plate reader (Multiskan Ex, Thermo Fisher Scientific) at a wavelength of 492 nm. Each condition was analyzed in triplicate. CellTiter Glo (Promega) was used in some experiments. According to protocol's instruction, 100 μl CellTiter Glo reagent was added into each well, the plates were briefly mixed by an orbital shaker and incubated for 10 min at room temperature. Luminescence was recorded by the Sparke multiplate reader (Tecan, Männedorf, Switzerland). Each variant group was performed in triplicate wells.

### Progenitor Cell Assays

1 x 10^5^ AML cells were resuspended in 100 μl 10% FBS/Iscove's modified Dulbecco's medium and cultured in 1 ml of semisolid methylcellulose medium supplemented with cytokines (StemMACS HSC-CFU lite with Epo, Miltenyi, Teterow, Germany) according to the manufacturer's specifications. Leukemic colony forming units (CFU- L) were scored after 10–12 days of incubation in standard conditions.

### Apoptosis

Apoptosis was measured with Annexin-V-FLUOS Staining Kit (Roche, Basel, Switzerland) according to the manufacturer's instructions. Briefly, 1 x 10^6^ cells were resuspended in 100 μl of Incubation Buffer, stained with 2 μl of FITC-conjugated Annexin-V and 2 μl of propidium iodide (PI) for 10 min at room temperature and analyzed by flow cytometry (AccuriTM C6, BD, Franklin Lakes, NJ, USA) and FCS Express 4 Software, De Novo Software, Glendale, CA, USA).

### Active Caspase-3 Expression

Cells were treated with a Fixation/Permeabilization kit (BD) for intracellular staining and incubated with anti-active Caspase-3 PE antibody (#550821) (BD), according to standard protocol and analyzed by flow cytometry as described.

For immunofluorescence staining, cells were fixed in 4% paraformaldehyde, permeabilized with PBS/0.25% and stained with anti-Caspase-3 antibody (#700182) (Invitrogen, Carlsbad, CA, USA) and secondary antibody (#F0205) (Agilent Technologies). Stained cells were examined under fluorescence Axiovert microscope (Zeiss, Germany).

### Mitochondrial Membrane Potential (ΔΨm) Measurement

ΔΨm was investigated using the BD™ MitoScreen Kit (BD) according to the manufacturer's instructions. Briefly, 1 x 10^6^ cells were harvested, washed twice with PBS and incubated with JC-1 solution for 15 min at 37°C. JC-1 monomers or aggregates were analyzed by flow cytometry.

### Seahorse XF Cell Mito Stress Test

Cell Mito Stress Test (XF Cell Mito Stress Test Kit, Agilent Technologies) was performed following the standard protocol (37). Briefly, THP1 and OCI-AML3 were seeded at the concentration of 5 x 10^5^/ml in a 24-well plate and treated with increasing doses of DEN. After 24 h incubation, growth medium from each well was replaced by pre-warmed assay medium (pH: 7.4) and counted. Cell were seeded at 50.000/well for THP1 and 150.000/well for OCI-AML3 in Seahorse 96-well plates coated with CellTak (BD Biosciences, San Jose, CA, USA) to facilitate attachment. OCR and Extracellular Acidification Rate (ECAR) were detected after injection of oligomycin (1 μM), Carbonyl cyanide-p-trifluoromethoxyphenylhydrazone (FCCP) (0.5 μM), and rotenone & antimycin (Rot/AA) combination (0.5 μM). In selected experiments, glycolytic proton efflux rate (glycoPER) was detected using the Seahorse XF Glycolytic Rate Assay (Agilent Technologies) for measuring glycolysis by injecting Rot/AA (0.5 μM) and 2-deoxy-D-glucose (2-DG) (50 mM). The assays were both performed by using the XFe96 analyzer (Agilent Technologies) and the data were analyzed by the Wave software (version 2.2.0, Seahorse Bioscience) after normalization.

### Migration Assay

Cell migration was tested using transwell assays (diameter 6.5 mm, pore size 8 μm Corning Costar, Corning, NY, USA) (38). Briefly, 100 μl RPMI 10% FBS containing 1 x 10^5^ cells was added to the upper chamber, while 600 μl medium w or w/o 150 ng/ml CXCL-12 (Meridian Life Science) was added to the bottom chamber. DEN was added to the upper or to the bottom chamber, in order to evaluate its priming or chemotactic activity, respectively. After overnight incubation at 37°C in 5% humidified CO_2_ atmosphere, inserts (upper chambers) were removed and cells transmigrated into lower chamber were recovered and counted. The number of migrating cells was counted with an inverted microscope (Nikon, Tokyo, Japan) using a 5 times magnification.

In some experiments, cells were pre-incubated for 4 h with DEN, which was washed out before migration assay.

### *In silico* Gene Expression Analysis

For *in silico* analysis, all calculations were performed using R version 3.6.1. CEL files raw data were normalized using Robust Multi-Array Average (RMA) and log_2_ transformed. In the TCGA datasets downloaded, the Affymetrix U133 Plus 2 was used to perform RNA-expression profiling, containing the following TAS2R transcripts probes: TAS2R1, TAS2R3, TAS2R4, TAS2R5, TAS2R7, TAS2R8, TAS2R9, TAS2R10, TAS2R13, TAS2R14, TAS2R16, TAS2R38, TAS2R39, TAS2R40, TAS2R41, TAS2R43, TAS2R45, TAS2R50. The differential expression levels of TAS2Rs were assessed in (1) 183 AML samples and (2) in association with clinical data, by the limma package (Bioconductor) (39). For all statistical analysis, we considered *p-*value < 0.05 and Benjamini–Hochberg adjusted *p*-values < 0.05.

### Statistical Analysis

Statistical analysis was performed using the unpaired two-tailed Student's *t*-test for comparisons between two groups or two-way ANOVA followed by *post-hoc* Dunnett's test for comparisons between three or more groups (GraphPad Prism 6.03, GraphPad Software, San Diego, CA, USA). Results are expressed as means ± SD, unless otherwise indicated. *P* < 0.05 was considered statistically significant.

## Results

### AML Cells Express Fully Functional TAS2Rs

**Figure 1:**
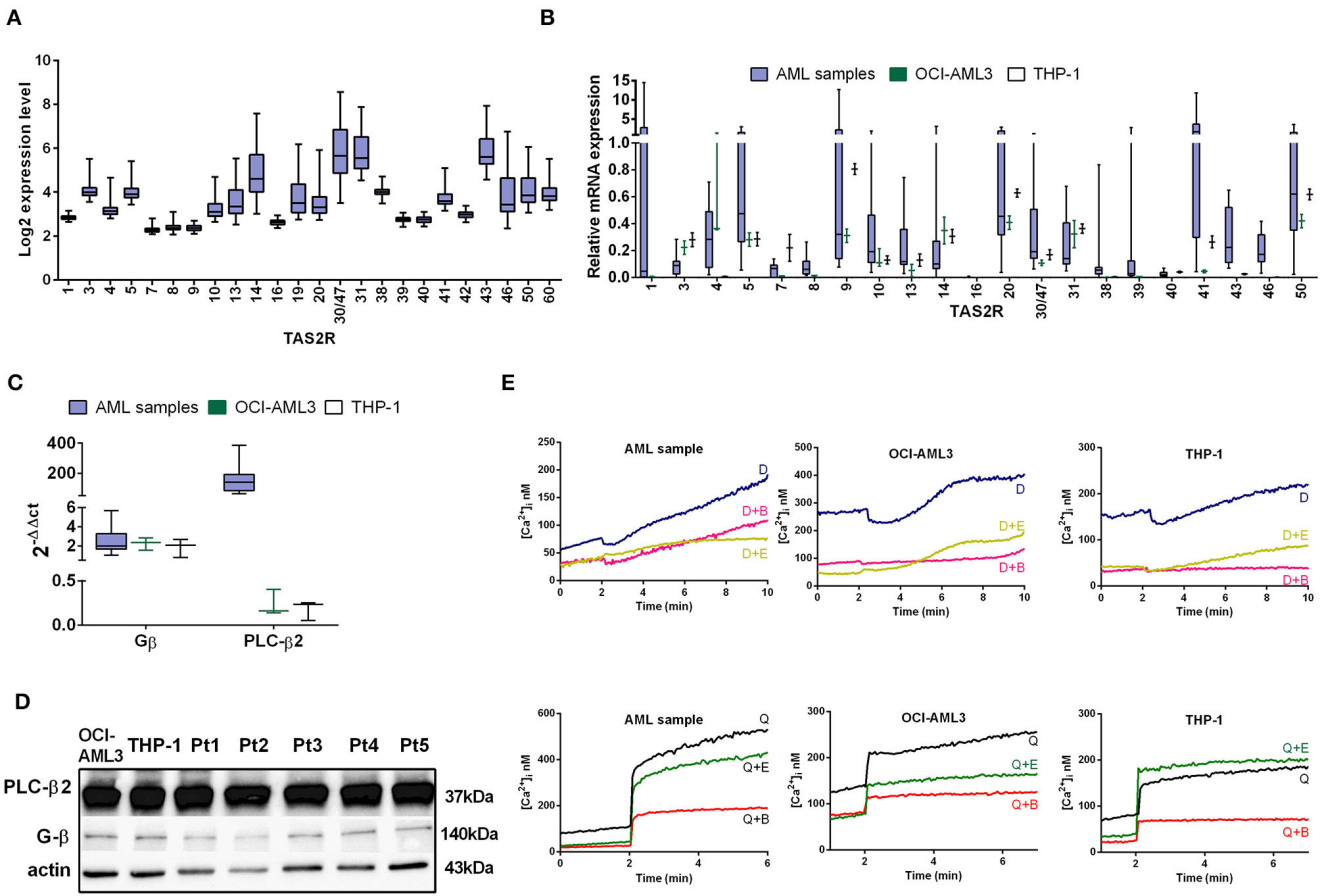
TAS2R expression in AML. **(A)** Box plots illustrate relative TAS2R mRNA expression obtained by GEP in primary AML samples (*n* = 61). **(B)** Box plot illustrate TAS2R mRNA expression analysis by qRT-PCR in primary AML samples (*n* = 13) and AML cell lines, THP-1 and OCI-AML3. Error bars refer to THP-1 and OCI-AML3 sample triplicates. Relative TAS2R expression levels were calculated using GAPDH as endogenous control and commercial cDNA pool as the reference sample was taken as 1 (2^−ΔΔCt^ method). Melt curve analysis confirmed the presence of a single PCR product and Ct values of expressed targets ranged between 25 and 30. **(C)** qRT-PCR analysis of TAS2R downstream targets in primary AML samples and AML cell lines. Relative target expression levels were calculated as described in **(A)**. Error bars in AML columns refer to the 13 analyzed samples. THP-1 and OCI-AML3 samples were analyzed in triplicates. **(D)** Western blot analysis of the downstream targets in two AML cell lines and five AML samples (Pt). Actin was shown as the loading control. **(E)** Ca^2+^ release in a representative AML sample and AML cell lines loaded with the Ca^2+^ indicator fura-2/AM and treated with 10 mM denatonium (indicated as D) or 75 μM quinine (indicated as Q) in presence of EDTA (indicated as E) or BAPTA-AM (indicated as B) buffer.

Based on the evidence of an extra-oral role of TAS2Rs, we investigated their expression and potential involvement in AML cell regulation. We analyzed gene expression microarray datasets of 61 samples of AML patients at diagnosis. First of all, we investigated the distribution of TAS2Rs transcript level among AML samples, highlighting a strong heterogeneity in the TAS2R expression genes (Figure 1A). Based on the log_2_ expression level, TAS2R transcripts were expressed in AML samples and in particular *TAS2R43, TAS2R13, TAS2R14, TAS2R30/47, TAS2R31/44* showed a higher level of TAS2R mRNAs than others. To validate the above results we downloaded gene expression profiling CEL files from the TCGA AML cohort consisting of 183 samples. The correspondence of gene expression level of TAS2Rs was very similar to our observations, as we have seen for TAS2R43, TAS2R10, TAS2R14 expression (Figure S1A). TAS2R mRNA expression levels were also assessed by qRT-PCR in an independent cohort of 13 AML samples and OCI-AML3 and THP-1 cell lines.

Despite the trend of the TAS2Rs expression level were similar in the 61 AML and in the 183 *in silico* AML samples, the qRT-PCR confirmed the trend for only 6 of the 21 TAS2Rs tested but showing the positivity for almost all TAS2Rs (Figure 1B). This discrepancy is probably due to different size of highly heterogeneous analyzed samples.

As GPCRs, TAS2Rs work together with gustducins, a class of taste receptor-specific G proteins, stimulating, through PLC-β2 activation, a signaling cascade leading to the calcium release from intracellular stores (40). Thus, we found that AML cells and cell lines expressed the β-gustducin and the PLC-β2, at mRNA and protein level, indicating that the necessary factors for canonical bitter taste signaling pathway are also present (Figures 1C,D). We noted that PLC-β2 mRNA was expressed at lower amounts in AML cell lines as compared to the AML primary cells (Figure 1C), while PLC-β2 protein expression levels seemed to be similar. This suggested the existence of some mechanism/s of PLC-β2 mRNA upregulation in AML primary samples and/or selective post-transcriptional events modulating PLC-β2 expression levels.

We next stimulated OCI-AML3 and THP-1 cell lines with three bitter compounds denatonium, quinine, and chloroquine and we determined the activity of TAS2Rs by analyzing calcium mobilization after stimulation. A rapid increase in calcium activity was seen in response to denatonium and quinine using the fura-2/acetoxymethyl ester (fura-2/AM) assay (Figure 1E and Figure S1B), while the response to chloroquine was almost null (not shown). To ascertain whether the measured increase of intracellular calcium was due to store-operated calcium release, as expected from TAS2R activation, or extracellular calcium influx we performed the same experiments in the presence of BAPTA-AM or EDTA. Figure 1E shows that, while both chelating agents reduce basal intracellular calcium levels, only BAPTA-AM, which is known to obliterate calcium present in intracellular stores, leads to a loss of calcium release into the cytosol following stimulation with denatonium and quinine. However, we observed a slight increase of calcium release also in presence of BAPTA-AM in AML primary samples after stimulation with denatonium, suggesting an involvement of other TAS2R-independent pathways as well (41).

Taken together, these results demonstrate that AML cells express functional TAS2Rs.

### TAS2R Expression Levels Correlate *in silico* With Some AML Clinical Parameters

**Figure 2:**
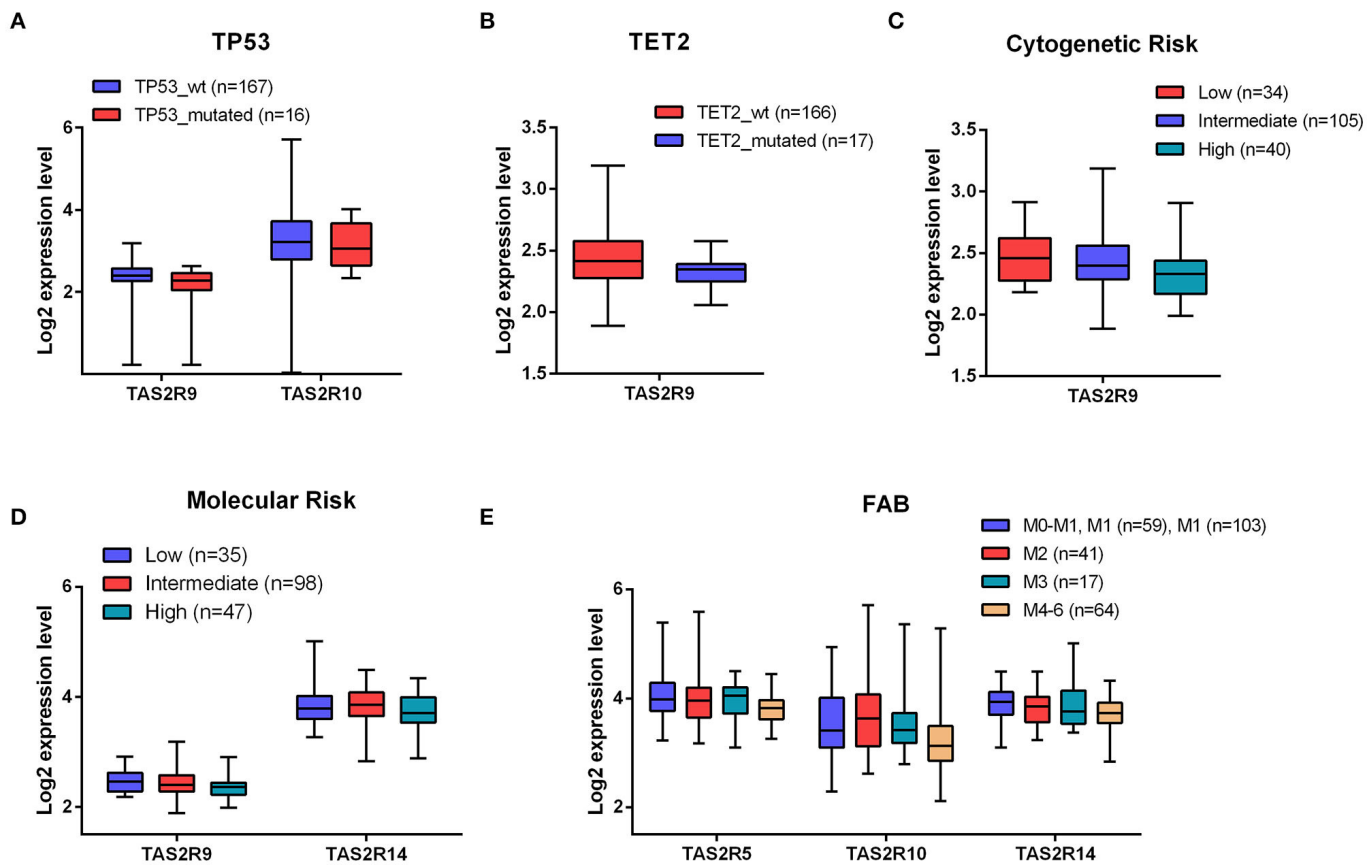
Association between TAS2R transcript levels and clinical parameters in AML patients. **(A,B)** Boxplots of different expression level of TAS2R10 and TAS2R9 in *TP53* and *TET2* mutated and wild-type samples; **(C–E)** Boxplots of the indicated TAS2Rs based on Cytogenetic molecular and FAB clinical features. Statistical analysis were performed by Student's *t*-test and Benjamini–Hochberg adjusted *p*-values correction.

To corroborate the significance of TAS2R expression in AML, we investigated a potential relationship between TAS2R transcript levels and clinical parameters in AML patients. To this aim, we performed an association analysis on 183 AML samples from a public database for which clinical data were available. Considering the recurrently mutated genes *TP53, FLT3, NPM1, DNMT3A, TET2, RUNX1, IDH1, IDH2* in AML, we found a statistically significant association between *TP53* mutated samples and a lower expression of TAS2R9 (*p*-value = 0.008) and TAS2R10 (*p*-value = 0.03) and between *TET2* mutated samples and a lower expression of TAS2R9 (*p*-value = 0.04) (Figures 2A,B). By analyzing the association between TAS2R expression level and the cytogenetic and molecular risk, we found that a low expression of TAS2R9 was significantly associated with both cytogenetics (*p*-value = 0.005) and molecular poor risk (*p*-value = 0.02) (Figures 2C,D) and a low expression of TAS2R14 (*p*-value = 0.02) was associated with a poor molecular risk (Figure 2D). Considering FAB classification, we also observed a significant association between a reduction in mRNA expression level of TAS2R10 (adjust BH *p*-value = 0.007), TAS2R5 (adjust BH *p*-value = 0.03), and TAS2R14 (adjust BH *p*-value = 0.045) and a more differentiated status of AML blast (Figure 2E). Any significant association was found between TAS2R expression levels and other parameters as age, sex, and or blast percentage in the BM. These data indicate that the expression of some TAS2Rs may be not randomly clustered with some relevant cell-intrinsic alterations of AML cells, providing the rationale for better investigating the functional relevance of TAS2Rs stimulation in AML cells.

### TAS2R Stimulation by DEN Alters the Expression of Genes Involved in AML Cell Function

**Figure 3:**
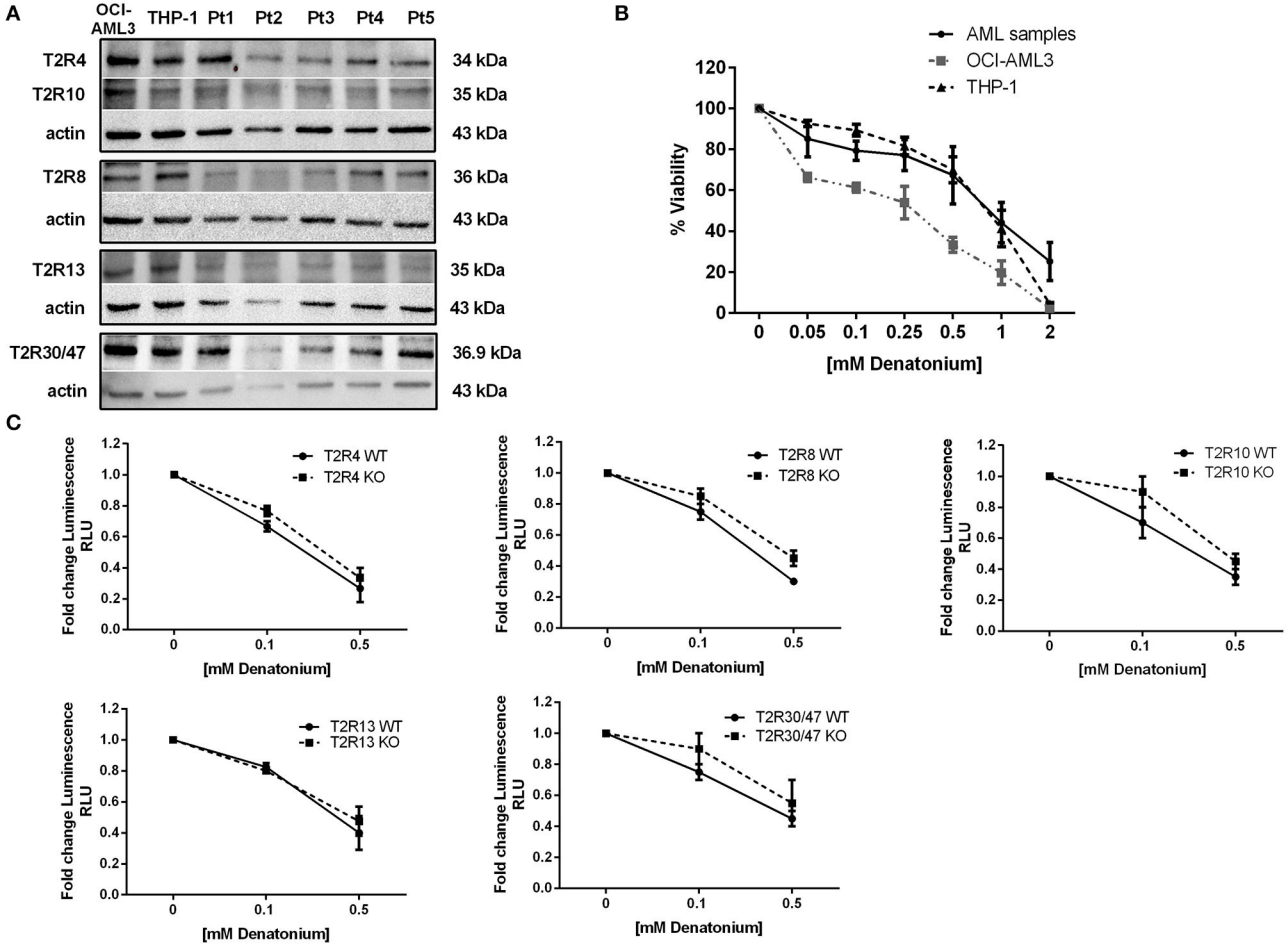
Denatonium affects leukemia cell viability. **(A)** Western blot analysis of the denatonium-sensitive T2Rs in two AML cell lines and five AML samples (Pt). Actin was shown as the loading control. **(B)** Cell viability detected by CellTiter 96 Aqueous One Solution assay in AML samples (*n* = 7) and AML cell lines (*n* = 3 for each cell line) treated for 48 h with increasing doses of DEN. **(C)** Cell viability detected by CellTiter-Glo assay in THP-1 wild type and in T2R4, T2R8, T2R10, T2R13, and T2R30 knockout THP-1 (*n* = 3 for each cell line).

To test the TAS2R function, we decided to use DEN as a model compound since, compared to quinine, DEN targets fewer TAS2Rs that could be downregulated in AML patients. DEN exerts their effects by activating 9 out 25 TAS2Rs (42), 5 of which (T2R4, T2R8, T2R10, T2R13, and T2R30/47) are expressed also at protein level both in AML samples and in AML cell lines (Figure 3A). We tested the DEN effective/not toxic (43) dose by exposing primary AML cells and OCI-AML3 and THP-1 cell lines to increasing doses of DEN and analyzed cell viability. After 48 h of exposure, DEN reduced both cell line and AML cell viability in a dose-dependent manner (Figure 3B). However, due to the high grade of redundancy in the expression of DEN-sensitive TAS2Rs, we could not identify a unique candidate in charge of the observed effect by using knockdown cells (Figure 3C).

**Figure 4:**
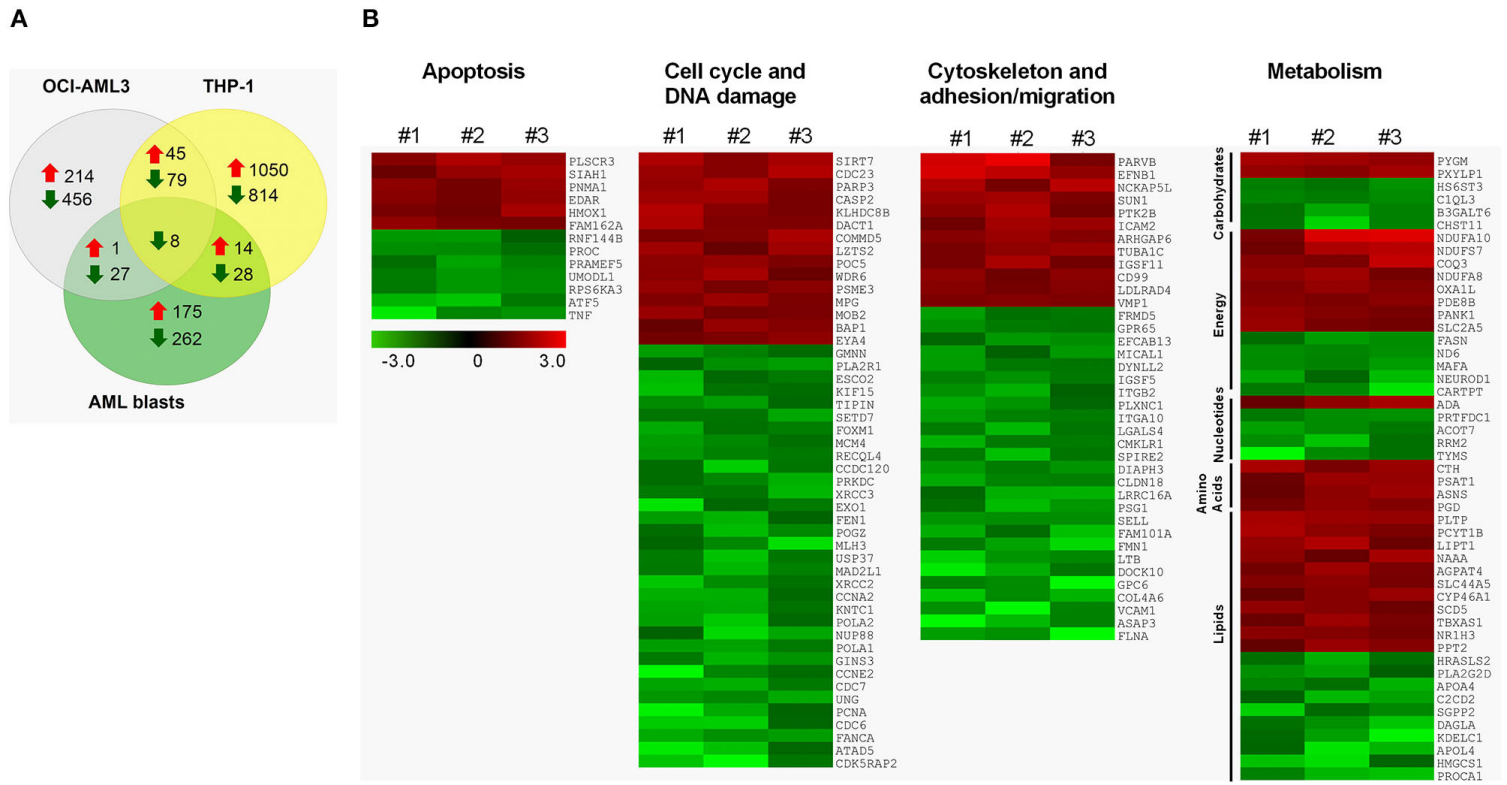
Transcriptomic alterations induced by DEN in AML. **(A)** The number of upregulated and downregulated genes after 24 h of denatonium exposure in primary AML cells (0.5 mM), OCI-AML3 (0.1 mM), and THP-1 (0.5 mM) cell lines. **(B)** Heatmap of differentially expressed genes involved in apoptosis, cell cycle and DNA damage, cytoskeleton, cell adhesion and migration, and metabolism, following exposure to denatonium in three primary AML sample. Columns represent ratios between DEN- and vehicle-treated cells for each case. Color changes are quantified by the scale bar. Genes are ranked according to their fold change.

To identify the cellular processes regulated by TAS2Rs in AML cells, we performed GEP after *in vitro* activation of the TAS2R pathway by DEN. Overall, DEN induced the upregulation of 190, 260 and 1109 genes and the downregulation of 325, 570 and 929 genes in primary AML, OCI-AML3 and TPH-1 cells, respectively (Figure 4A). Although several genes were uniquely altered in the analyzed cells, interestingly, a core transcriptional program of 45 upregulated and 87 downregulated genes was shared between OCI-AML3 and THP-1 cells (Figure 4A and Table S4). Among them, 8 genes were significantly downregulated also in primary cells (Figure 4A and Table S4). Downregulated transcripts in AML cells were significantly enriched for genes involved in the cell cycle and DNA damage, cytoskeletal function, cell adhesion and migration, and pyrimidine metabolism (Figure 4B and Table S5). Moreover, DEN treatment altered the expression of genes involved in apoptosis, carbohydrate, energy, amino acid, and lipid metabolism in AML cells (Figure 4B). Among apoptosis-related genes, *TP53INP1, RNF130, CFLAR, TNFSF10* were upregulated and *AIFM2* was downregulated in both OCI-AML3 and THP-1 (Table S4). The two cell lines also shared the deregulation of genes involved in cytoskeletal function, cell adhesion and migration (upregulated: *PIK3R5;* downregulated: *TUBA8, FSCN1, HMMR, RHOF, EMD*) and cell cycle/DNA damage (upregulated: *FRY, LIG4, AKAP9;* downregulated*: ORC1, SPDL1, NCAPG2, RAD54B, NCAPG, KIF22, PBK, BTG3, CDC25A, CDC45, PLK1, CDC20, CDCA3, CCNF, CCND1, CENPV, FANCG*). Differentially expressed transcripts in the tested cell lines were enriched for genes involved in cell cycle, DNA damage and glycolysis (Tables S6, S7). According to pathway analysis, cytoskeletal function, cell adhesion and migration, fatty acid biosynthesis, and glycolysis were significantly enriched in OCI-AML3 (Table S6), while THP-1 cells showed a preferential enrichment for transcriptional alterations targeting metabolism-related genes, including bioenergetics pathways (glycolysis and mitochondrial respiration), biosynthetic processes (purine, nucleotides, amino acids), and lipid metabolism (fatty acid, cholesterol) (Table S7). Gene set enrichment analysis (GSEA) corroborated pathway analysis (Figure S2).

Overall, our data suggest that the TAS2R pathway induction by DEN deregulates relevant cellular processes in AML cells, including cell cycle, survival, migration, and metabolism.

### DEN Inhibits AML Cell Proliferation and Clonogenic Efficiency

**Figure 5:**
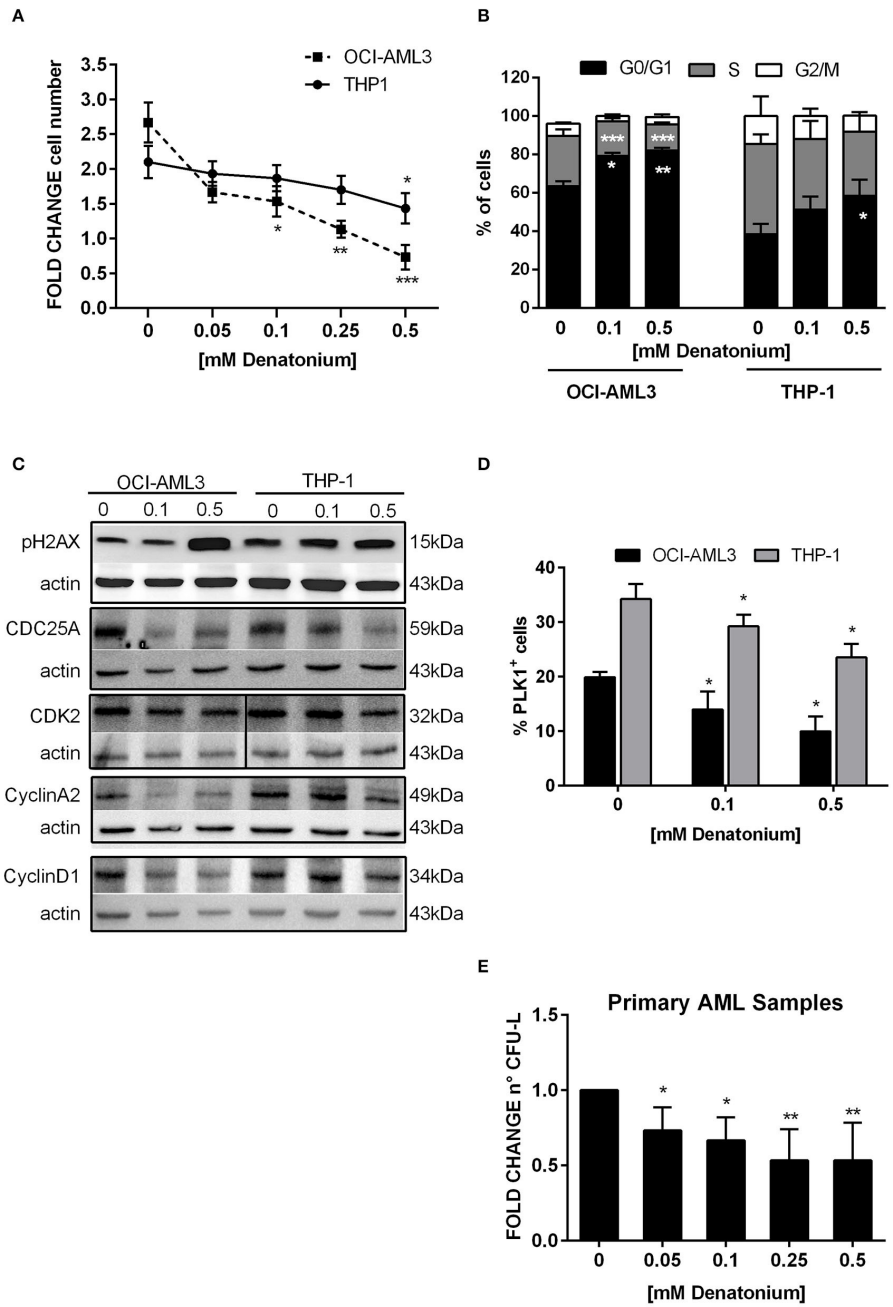
DEN stimulation inhibits AML cell proliferation and clonogenic ability. **(A)** Cell proliferation determined by CellTiter 96 Aqueous One Solution assay and normalized to time 0 in THP-1 (*n* = 3) and OCI-AML3 (*n* = 3) cultured for 48 h with increasing doses of DEN. **(B)** Histograms display cell cycle phase distribution in AML cell lines after 48 h of DEN exposure (*n* = 5). **(C)** Western blot analysis of the indicated proteins in two AML cell lines treated with the indicated doses of DEN for 24 h. Actin was shown as the loading control. **(D)** Percentage of PLK1^+^ cells analyzed by flow cytometry in AML cell lines treated with the indicated doses of DEN for 24 h (*n* = 4). **(E)** Histograms indicate the fold-change of the CFU-L obtained from primary AML cells cultured in semisolid medium in the presence of cytokines and increasing concentration of DEN (*n* = 7). The mean number of colonies in control was 22 ± 12 and taken as 1. Data are expressed as mean ± SEM in **(A–D)** and as mean ± SD in **(E)**. Statistical analysis was performed by using ANOVA followed by Dunnett's multiple comparison test with untreated group as control. *p < 0.05; **p < 0.01; ***p < 0.001.

Consistently with the molecular studies, exposure to non-toxic doses of DEN exerted an antiproliferative effect on AML cells *in vitro*. OCI-AML3 and THP-1 proliferation was significantly reduced after 48 h of DEN treatment, (Figure 5A). Cell cycle analysis showed that the inhibitory effect of DEN was mainly due to a G0/G1-phase arrest and a concomitant S-phase decrease (Figure 5B).

To substantiate these data, Western blot analysis was used to validate the expression of cell cycle proteins, which GEP analysis indicated as deregulated at the mRNA level. The expression of cyclin D1 and cyclin A2, which normally increased during G1 (44) and S phase progression (45) respectively, was significantly reduced following DEN-treatment in both cell lines (Figure 5C). In parallel, the phosphatase cell division cycle 25 homolog A (CDC25A), able to activate G1/S cyclin-dependent kinase 2 (CDK2) (46), decreased in DEN-treated cells. Instead, CDK2 was downregulated to a lesser extent, according to an inactivation mechanism based on cyclin level oscillation during the cell cycle (47). Consistently with a cell cycle arrest, the expression of a typical mitotic protein, i.e., polo-like kinase 1 (PLK1), whose activation relies on Cyclin A2-Cdk activity levels (48), was significantly inhibited in DEN-treated cells (Figure 5D). GEP analysis also highlighted that differentially expressed transcripts were enriched for genes involved in DNA repair (e.g*., RAD54B, XRCC*3, and *FANCG* showed reduced expression in both cell lines after treatment). Accordingly, protein expression analysis showed an increase in the phosphorylated form of the histone 2AX (pH2AX) following DEN treatment in AML cell lines (Figure 5C), indicating an increase in DNA damage, probably due to impaired DNA repair activity.

To evaluate the TAS2R stimulation effect on the growth properties of leukemic progenitor cells, colony growth was determined by CFU assay using increasing doses of DEN. Non-toxic doses of DEN significantly inhibited CFU-L formation already at the lowest dose and up to 50% inhibition at the highest doses (Figure 5E).

Overall, these results support GEP data, suggesting that AML cells respond to TAS2R signaling pathway activation at low doses of DEN by accumulating DNA damage and reducing proliferative and clonogenic potential.

### Exposure to High Doses of DEN Induces AML Cell Apoptosis

**Figure 6:**
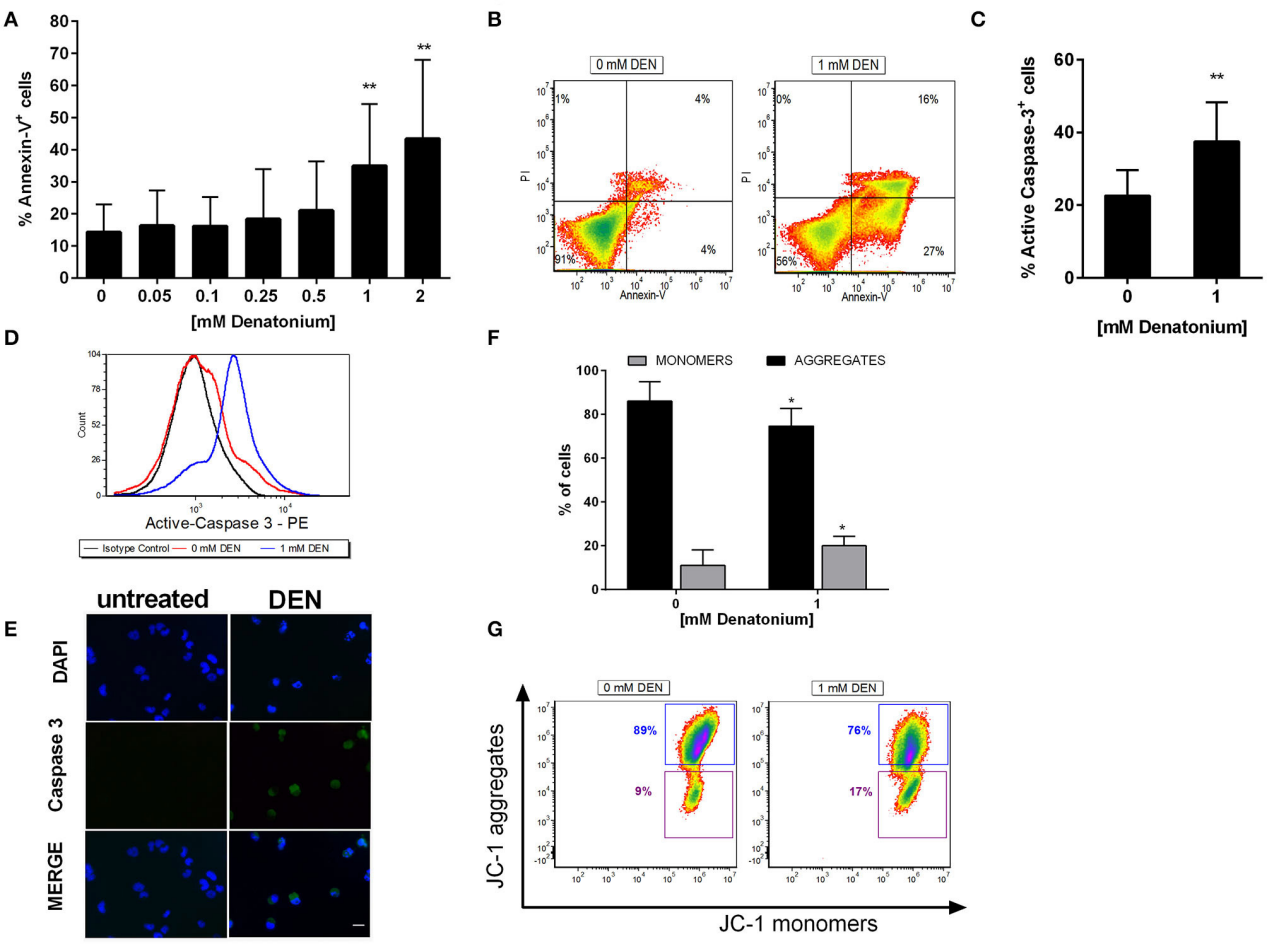
DEN induces AML cell apoptosis. **(A)** AML cells (*n* = 10) were treated for 48 h with increasing doses of DEN. Annexin V/PI staining was used to detect apoptosis. **(B)** Representative FACS analysis of apoptosis after treatment with 1 mM DEN. **(C)** FACS analysis of active caspase-3 expression in AML cells after treatment with 1 mM DEN for 48 h (*n* = 6). **(D)** Representative overlay histograms of Active caspase-3 expression. **(E)** Immunofluorescence analysis of activated caspase-3 (green) after 48 h of treatment with 1 mM DEN. Nuclei were counterstained with DAPI (blue). 40X magnification, scale bar 20 μm. **(F)** FACS analysis of mitochondrial membrane potential of AML cells after 48 h of exposure to 1 mM DEN. The histogram shows the percentage of JC-1 aggregates (cells emitting red fluorescence in the FL-2 channel) and JC-1 monomers (cells emitting green JC-1 detected in the FL-1 channel) (*n* = 6). **(G)** Representative dot plots of JC-1 staining. Data are expressed as mean ± SD. Statistical analysis was performed by using ANOVA followed by Dunnett's multiple comparison test with untreated group as control and by Student's *t*-test. *p < 0.05; **p < 0.01.

Since GEP data showed modulation of genes involved in apoptosis and exposure to high doses of DEN induced a significant reduction of AML cell viability, we evaluated the potential cytotoxic effect of TAS2R stimulation. Thus, we treated AML cells (*n* = 10), OCI-AML3 and THP-1 cells with increasing doses up to 2 mM DEN and analyzed the cells after Annexin-V/PI staining. As shown in Figures 6A,B, necrosis induction was not observed, as demonstrated by the absence of a PI^+^/Annexin-V^−^ cell population. Conversely, there was a 3-fold increase of the percentage of AML apoptotic cells at the high doses of agonist compared to control. The same results were obtained in OCI-AML-3 and THP-1 cells (Figures S3A,B). To better characterize apoptosis after DEN treatment, we evaluated caspase cascade activation by analyzing the expression of caspase 3 active form. Flow cytometry (Figures 6C,D) and immunofluorescence (Figure 6E) revealed an increased expression of active-caspase 3 in AML cells after DEN exposure. Similar results were obtained in AML cell lines (Figure S3C). To confirm mitochondrial involvement in apoptosis after DEN treatment, we stained AML cells with JC-1 dye, which accumulates as aggregates or monomers in healthy or damaged mitochondria, respectively. As shown in Figures 6F,G, 48 h of DEN exposure reduced ΔΨm in treated compared to untreated AML cells, as demonstrated by the 2-fold increase of JC-1 monomers and the concomitant significant decrease of JC-1 aggregates. Similar results were obtained in AML cell lines (Figures S3D,F). The induction of apoptosis is probably independent of reactive oxygen species (ROS) activation since DEN was unable to induce ROS production in the tested AML cells (Data not shown).

Taken together, our results indicate that AML cells undergo apoptosis after TAS2R stimulation by high DEN dose through caspase and mitochondria pathway activation.

### DEN Alters AML Cell Mitochondrial Metabolism

**Figure 7:**
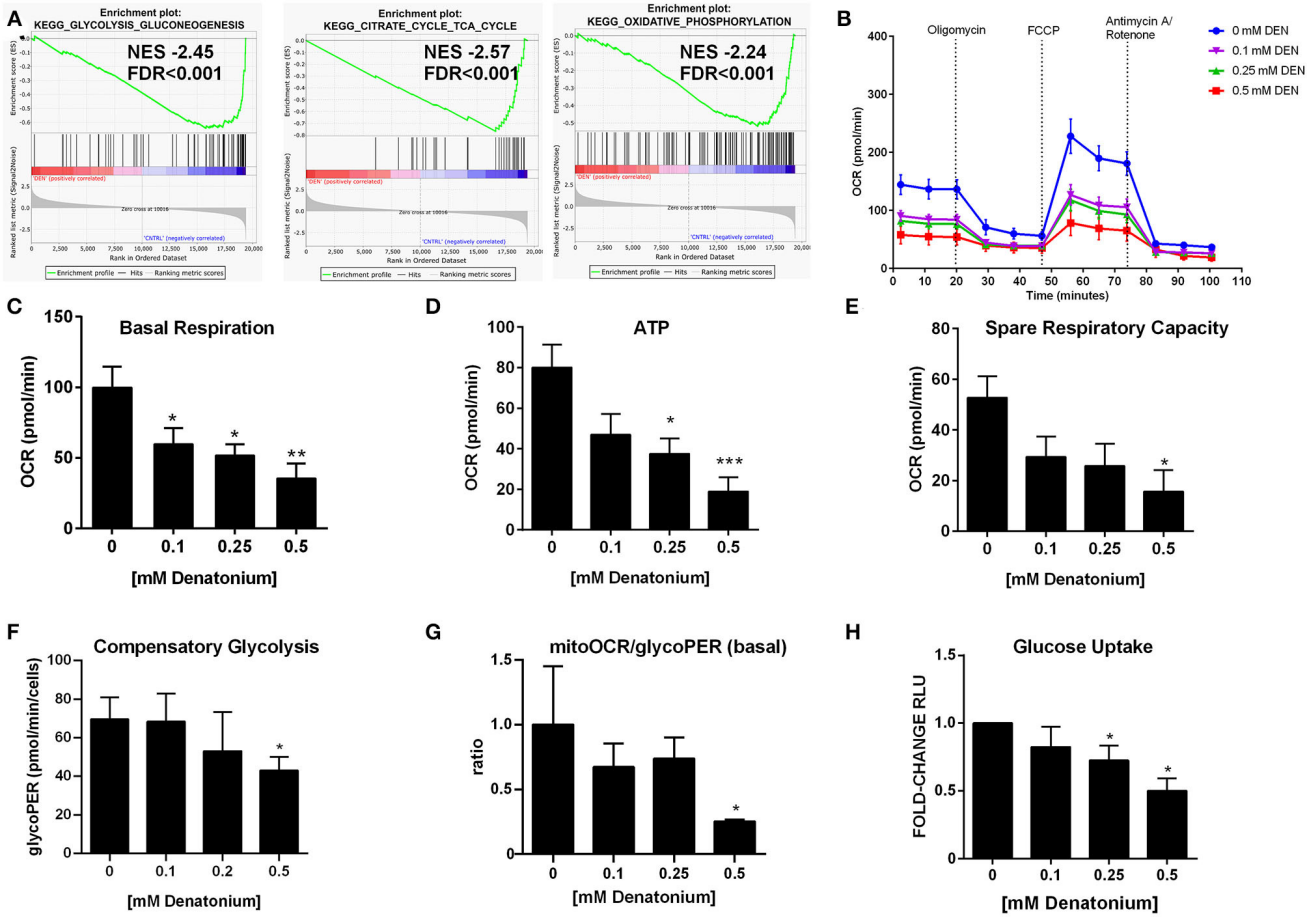
DEN alters mitochondrial bioenergetics. **(A)** Downregulation of gene signatures of glycolysis, citrate cycle and OXPHOS defined by GSEA in THP1 cells after 24 h of exposure to DEN. **(B)** Oxygen consumption rate (OCR) profile plot obtained by the Seahorse Cell Mito Stress Test in the THP-1 cell (*n* = 4) after 24 h of exposure to increasing doses of DEN. **(C)** Basal respiration. **(D)** ATP-linked respiration. **(E)** Spare capacity. **(F,G)** Quantitative data of compensatory glycolysis and ratios of mitochondrial OCR to glycoPER calculated using Seahorse XF Glycolytic Rate assay (*n* = 3). **(H)** Measurement of glucose uptake in THP-1 cells after 24 h exposure to increasing doses of DEN (*n* = 5). Untreated cells (193,001 ± 44,454 RLU) were used as a reference and set as 1. Data are expressed as mean ± SEM. Statistical analysis was performed by using ANOVA followed by Dunnett's multiple comparison test with untreated group as control. *p < 0.05; **p < 0.01; ***p < 0.001. NES, normalized enrichment score; FDR, false discovery rate.

GSEA indicated a significant enrichment for a gene signature of glycolysis and gluconeogenesis/citric acid cycle/oxidative phosphorylation (OXPHOS) in untreated compared to DEN-treated leukemia cells (Figure 7A and Figure S4A), suggesting an alteration in cellular bioenergetics after DEN exposure. To confirm GSEA and GEP data, we measured cellular bioenergetics following mitochondrial metabolic stress. As shown by Oxygen Consumption Rate (OCR), DEN treatment decreased both basal and maximal mitochondrial respiration (Figure 7B and Figure S4B). Specifically, mitochondrial basal respiration was significantly disrupted, as evidenced by the basal respiration drop of more than 50% (Figure 7C and Figure S4C). These results are also consistent with a 2-fold decrease in ATP-linked respiration in DEN-treated cells, in both THP-1 and OCI-AML3 (Figure 7D and Figure S4D). Respiratory spare capacity represents the reserve capacity of a cell to generate ATP via OXPHOS following an increased energy demand. This mitochondrial reserve capacity was reduced by more than 50% in DEN-treated compared to untreated cells (Figure 7E and Figure S4E). These data suggest that DEN treatment decreased mitochondrial (mt)OXPHOS and makes AML cells more prone to oxidative and metabolic stress due to decreased substrate availability or mitochondrial dysfunction. Concomitantly, to further characterize the relative utilization of glycolysis and OXPHOS after DEN treatment, we performed the Glycolytic Rate Assay, measuring basal glycolytic rates and compensatory glycolysis following mitochondrial inhibition. Despite no difference in terms of basal glycolysis (Figures S5A,C), high doses of DEN (0.5 mM) increased the percentage of proton efflux rate (PER) from glycolysis in THP-1 (Figure S5B). Consistent with the dropped level in the basal respiration detected by Mito Stress assay, these data suggest that glycolysis, rather than mitochondrial-derived CO_2_, is the main contributor to extracellular acidification and PER. However, at the same concentration, DEN is able to markedly impair the compensatory glycolysis achieved by the cells after blockage of mitochondrial ATP production (Figure 7F). In OCI-AML3, DEN showed a lower impact in terms of glycolytic activity (glycoPER) (Figures S4F, S5D). Importantly, as indicated by mitoOCR/glycoPER basal ratio (Figure 7G and Figure S4G), DEN decreased of 70% the rate of acidification in both OCI-AML3 and THP-1 due to mitochondrial metabolism, indicating that DEN shifts the bioenergetics profile of the cells from OXPHOS toward aerobic glycolysis, likely impaired under stress.

We hypothesized that the observed reduction of both mtOXPHOS and glycolysis could also be due to reduced glucose uptake after DEN exposure. As shown in Figure 7H and Figure S4H, we observed a significant decrease in glucose uptake in DEN-treated compared to untreated cells.

Overall, the results suggest that DEN-treated cells are unable to maintain basic cell function and to use both mitochondrial respiration and glycolysis, showing a defective metabolic and quiescent phenotype.

### DEN Exposure Inhibits AML Cell Motility

**Figure 8:**
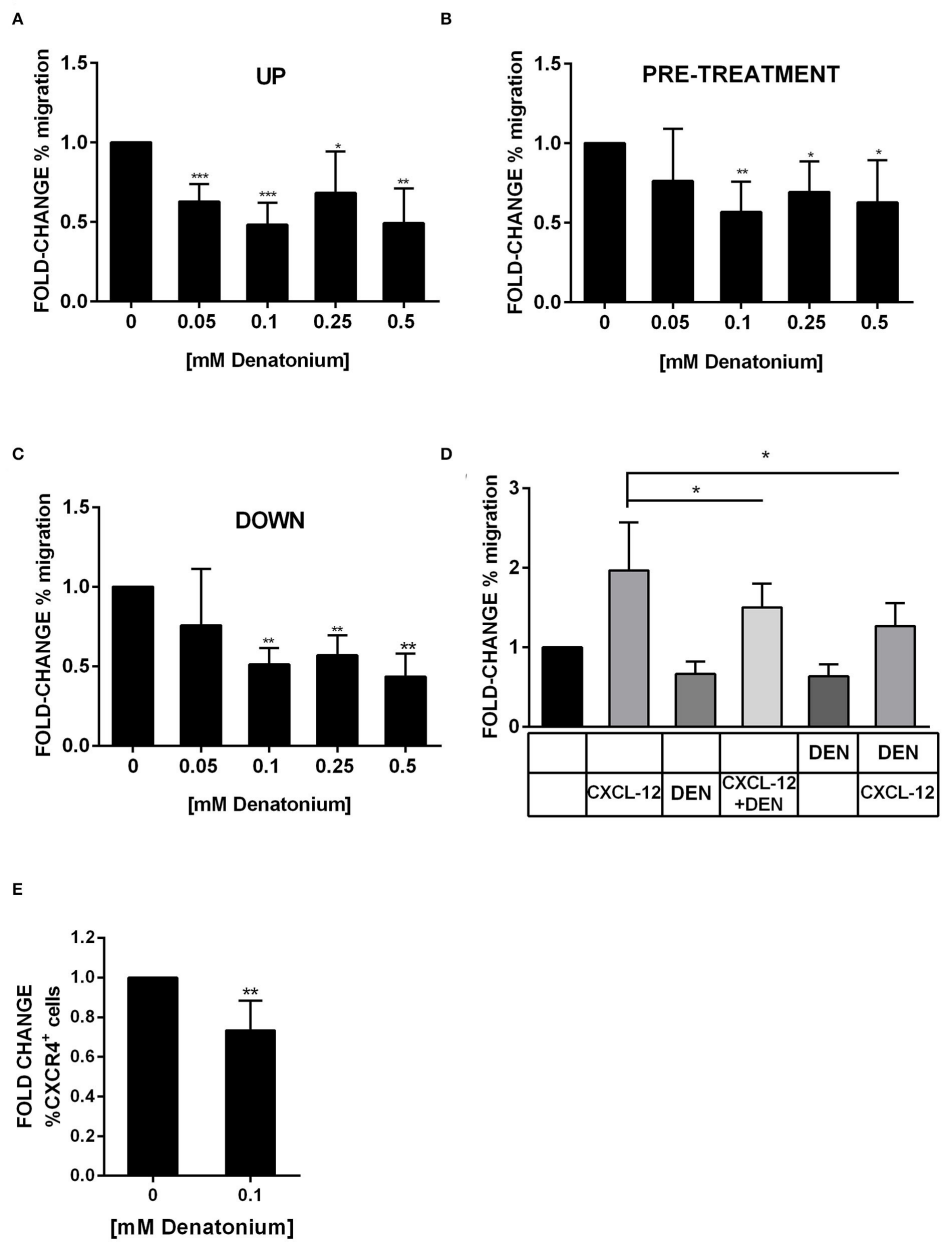
DEN stimulation inhibits AML cell migration. Results of AML cell migration in transwell assays were shown as fold-change of the percentage of migration in comparison with the untreated condition set as 1. **(A)** Spontaneous migration of AML cells in presence of increasing doses of DEN in the upper chamber of transwell (*n* = 6). The percentage of migrating cells in control samples was 29.5 ± 6.3 SD. **(B)** Spontaneous migration of AML cells after a pre-treatment of 4 h with increasing doses of DEN (*n* = 8). The percentage of migrating cells in control samples was 34.3 ± 11.3 SD. **(C)** Migration of AML cells toward a gradient of DEN in the lower chamber of transwell (*n* = 5). The percentage of migrating cells in control samples was 32.0 ± 9.7 SD. **(D)** The histogram shows the effect of the presence of 0.1 mM DEN in the upper or the lower chamber on the CXCL-12 induced chemotaxis (150 ng/ml). The percentage of migrating cells in control samples was 34.1 ± 12.8 SD (*n* = 5). **(E)** CXCR4 expression analyzed by flow cytometer after overnight treatment with DEN (*n* = 6). The percentage of CXCR4^+^ cells in untreated samples was 23.6 ± 13.1 SD. Data are expressed as mean ± SD. Statistical analysis was performed by using ANOVA followed by Dunnett's multiple comparison test with untreated group as control and by Student's *t*-test. *p < 0.05; **p < 0.01; ***p < 0.001.

GEP data suggested that AML cell motility was affected by DEN treatment. Thus, we tested the DEN effect on AML cell migratory capacity by using the transwell system. When increasing doses of DEN were added to the transwell upper chamber, the direct exposure to DEN reduced by about 50% the spontaneous migration of primary AML cells, even at the lower dose (Figure 8A). Similar results were obtained in AML cell lines (Figure S6A). Moreover, the inhibition persisted even if the stimulus was removed from the medium. Indeed, pre-treatment with DEN of both AML cells (Figure 8B) and cell lines (Figure S6B) reduced of 30% their spontaneous migration. Furthermore, the motility inhibition was even remarkable in the presence of a DEN gradient, obtained through the addition of DEN to the transwell lower chamber (Figure 8C and Figure S6C). The CXCL-12-CXCR4 axis, the key mediator in hematopoietic stem cell migration, is exploited by AML cells and regulates their trafficking in the BM microenvironment. Therefore, we wondered if DEN exposure affected the CXCL-12-CXCR4 axis. In presence of DEN, AML cells reduce by about 30% their migration toward the chemoattractant agent CXCL-12 (Figure 8D and Figures S6D,E). Moreover, DEN exposure induced a 0.7 fold-change of the CXCR4 surface expression (Figure 8E and Figure S6F).

Next, we investigated whether DEN altered AML cell interaction with extracellular matrix components, which play a key regulatory role in cell motility and trafficking. Using fibronectin-coated culture wells, we found that DEN treatment did not induce a significant modulation of AML cell adhesion capacity and did not affect the expression of CD49e, CD49d, and CD29 integrins (data not shown).

These results suggest that exposure to DEN attenuates leukemia cell migration likely through the inhibition of the CXCR4-CXCL-12 axis rather than AML cell adhesion to the extracellular matrix *in vitro*.

## Discussion

In the present work, for the first time, we demonstrated that TAS2Rs are involved in the regulation of leukemia cell functions.

TAS2Rs are known for their primary role as a central warning signal to induce aversion toward noxious or harmful substances. Nevertheless, the increasing amount of evidence about their extraoral localization has suggested a wider function in sensing microenvironment, also in cancer settings.

Our data, obtained from AML cell lines and primary AML cells extend our knowledge about the expression of TAS2Rs to the field of the hematological malignancies.

We showed that AML cells express TAS2Rs, coupled with the canonical signaling components such as the β-subunit of gustducin and PLC-β2. In bitter taste signal transduction, β/γ subunits of G-proteins are known to initiate the dominant branch of the pathway, via PLC-β2 activation and calcium release (40, 49, 50). To substantiate TAS2R functionality in AML cells, we stimulated AML cells with DEN and quinine, two putative TAS2R ligands, commonly used for TAS2R activation (11, 41, 51–53) and we found that both significantly mobilized intracellular calcium. TAS2R activation is known to increase intracellular calcium due to store-operated calcium release (54). Accordingly, when calcium present in intracellular stores had been obliterated, we found a loss of calcium release following stimulation with denatonium and quinine. Although we cannot rule out a concomitant TAS2R-independent effect of bitter compounds in AML cells, i.e., via direct interaction with ion channels (41), these data indicate that, at least in part, TAS2R activation is involved in the effect of DEN and quinine on calcium mobilization, corroborating an on-target activity.

Supporting a potential functional role of TAS2Rs in AML cells, the expression of TAS2Rs has been significantly correlated in a large cohort of AML patients with some relevant biologic features, commonly used for diagnosis and risk stratification. In particular, we observed a significant modulation of some TAS2Rs in poor-prognosis AML groups, *TP53-*mut and *TET2*-mut patients, in line with the observed TAS2R level decrease in breast cancer cells with a more aggressive phenotype (18). Although far from being conclusive, our *in silico* correlative results support the idea of a potential TAS2R role in AML cell biology.

To identify the cellular processes regulated by TAS2Rs in AML cells, we performed a GEP analysis after exposure to the TAS2R agonist, DEN. A consistent number of genes were differentially expressed in AML cells following DEN treatment. Interestingly, pathway enrichment analysis indicates that diverse and relevant cellular processes, among which cell cycle, apoptosis, cell adhesion, migration, and metabolic activity are targeted by TAS2R pathway induction. The activation of these specific pathways was confirmed in functional *in vitro* experiments. All these pathways, only seemingly unrelated, concur to corroborate an “anti-proliferative” effect due to DEN treatment.

In healthy human airway smooth muscle cells and ovarian, prostate and breast cancer cell lines TAS2R-stimulation by DEN provides an anti-proliferative signal (17, 20, 55, 56). Accordingly, we found that stimulation with DEN inhibits AML cell proliferation. We also observed a DEN-dependent inhibitory effect on AML cell clonogenic capacity, a peculiar feature of leukemic cells, which is known to impact on prognosis (57, 58). However, the high grade of redundancy in the expression of TAS2Rs did not help to identify a unique candidate accounting for the TAS2R-mediated phenotype, also by using knockdown cells. Because it is known that an imbalance between cell proliferation and apoptosis is a hallmark in tumor cells (59, 60), we investigated the possibility that DEN could influence apoptotic mechanisms in AML cells. DEN exposure induces AML cell apoptosis through caspase and mitochondria pathway activation. Interestingly, we found that this effect is not observed in normal TAS2R-expressing CD34^+^ cells which are almost entirely unaffected (manuscript in preparation). These results suggest that DEN induces anti-tumor biological responses, and they are in agreement with recent reports suggesting that TAS2R activation, together with an anti-proliferative effect, induce apoptosis in breast, prostate, and ovarian cancer cells (17, 20). In this light, downregulation of some TAS2R, as observed in subgroups of patients, in particular in *TP53*- and *TET2*-mutated, might be a strategy adopted by AML cells to evade possible growth-suppressive effects, as demonstrated in breast cancer cells (18). However, due to the high AML heterogeneity, additional transcriptomic analysis on larger sample size and specific TAS2R-inactivation experiments are necessary, before drawing any meaningful conclusion.

We also found that DEN is able to alter mitochondria metabolism in AML cells. Changes in mitochondrial metabolism indicate adaptation to stress conditions and increased nutrient demand and could influence the cell-cycle progression and *vice versa* (61). Our data indicate that DEN-treated cells have significantly limited mitochondrial activity with a shift of the bioenergetic profile from OXPHOS to aerobic glycolysis, which, however, is likely impaired in stress conditions as also indicated by GEP data. Taken together, these data suggest that DEN concomitantly inhibits the mitochondrial OXPHOS and induce a “quiescent state” by arresting the cell-cycle progression.

The effects of DEN treatment on the migration of AML cells were also examined. Interestingly, DEN exposure mitigated both the spontaneous migration and the migration in the presence of a chemotactic gradient of CXCL-12. The observed inhibition of migration is possibly correlated with cell cycle arrest and Cyclin D1 and Cyclin A2 decrease. Indeed, beyond their role in cell cycle progression, Cyclin D1 and Cyclin A2/CDK2 complex are also involved in cellular structure organization and motility regulation (62, 63). Consistently, upon exposure to DEN, non-proliferating AML cells have reduced cell motility and migration, which may contribute to the AML cell anchorage in the protective BM niche and eventually generate resistance to therapy. A similar TAS2R-dependent phenotype has been already characterized in breast cancer cells (20). Our results are also in agreement with the previously reported role of TAS2R8 and TAS2R10 in abrogating migration of neuroblastoma cells (15).

Several natural bitter compounds, flavonoids and iso-flavonoids have displayed anti-cancer effects against various cancer types (25, 64–68) similar to DEN. Many of these compounds are recognized as TAS2R agonists (69, 70) but it is still unclear if the anticancer effects evoked by these bioactive compounds are mediated by TAS2R engagement or other indirect and unknown mechanisms. Despite the mechanism and the specific TAS2R involved in each process remains to be elucidated, our data suggests that “bitter” molecules present endogenously in the BM microenvironment, such as amino acids (71, 72), or extrinsic factors, such as drugs (42, 73), might interact with TAS2Rs and affect leukemia cell functions. Since, previous data (74) and our unpublished observations indicated that TAS2R were expressed also in healthy hematopoietic cells, we could speculate that TAS2R may represent a novel receptor-based pathway by which blood cells “taste” their microenvironment and respond to it accordingly.

In conclusion, our results in AML cells expand the observation of cancer TAS2R expression to the setting of hematological neoplasms and shed light on a role of TAS2Rs in the extrinsic regulation of leukemia cell functions.

## Data Availability Statement

The datasets presented in this study can be found in online repositories. The names of the repository/repositories and accession number(s) can be found below: the NCBI Gene Expression Omnibus (GSE149548).

## Ethics Statement

This research was approved by the Ethics Committee of Policlinico S. Orsola-Malpighi, University Hospital of Bologna and each individual gave written informed consent (Ethical Committee approval code: 147/2013/O/Tess).

## Author Contributions

VS and AC: study design and concept. VS, MCi, VP, GS, ML, SB, MP, ED, DF, and SO: data acquisition. VS and MCi: data analysis and interpretation, and manuscript preparation. AC, GM, FB, SM-F, EA, FD, and MCa: manuscript review. All authors read and approved the final manuscript.

## Conflict of Interest

The authors declare that the research was conducted in the absence of any commercial or financial relationships that could be construed as a potential conflict of interest.

## Abbreviations

TAS2R: bitter taste receptor
GPCR: G protein-coupled receptor
DEN: denatonium benzoate
BM: bone marrow
qRT-PCR: quantitative Real-time PCR
fura-2/AM: fura-2/acetoxymethyl ester
CDC25A: cell division cycle 25 homolog A
CDK2: cyclin-dependent kinase 2
PLK1: polo-like kinase 1
pH2AX: histone 2AX
GSEA: Gene set enrichment analysis
GEP: Gene Expression profiling
ROS: reactive oxygen species
CFU: colony-forming unit
OXPHOS: oxidative phosphorylation
OCR: Oxygen Consumption Rate
PER: proton efflux rate
FCCP: Carbonyl cyanide-p-trifluoromethoxyphenylhydrazone
ECAR: Extracellular Acidification Rate.
