# Corrigendum: Denatonium as a Bitter Taste Receptor Agonist Modifies Transcriptomic Profile and Functions of Acute Myeloid Leukemia Cells

**DOI:** 10.3389/fonc.2021.668460

**Published:** 2021-04-08

**Authors:** Valentina Salvestrini, Marilena Ciciarello, Valentina Pensato, Giorgia Simonetti, Maria Antonella Laginestra, Samantha Bruno, Martina Pazzaglia, Elena De Marchi, Dorian Forte, Stefania Orecchioni, Giovanni Martinelli, Francesco Bertolini, Simon Méndez-Ferrer, Elena Adinolfi, Francesco Di Virgilio, Michele Cavo, Antonio Curti

**Affiliations:** ^1^Department of Experimental, Diagnostic and Specialty Medicine, Policlinico S. Orsola-Malpighi University Hospital, University of Bologna, Bologna, Italy; ^2^Istituto Scientifico Romagnolo per lo Studio e la Cura dei Tumori IRCCS, Meldola, Italy; ^3^Laboratory of Experimental Oncology, IRCCS Istituto Ortopedico Rizzoli, Bologna, Italy; ^4^Department of Morphology, Surgery and Experimental Medicine, Section of Pathology, Oncology and Experimental Biology, University of Ferrara, Ferrara, Italy; ^5^Laboratory of Hematology-Oncology, IRCCS European Institute of Oncology, Milan, Italy; ^6^Department of Haematology, Wellcome Trust-Medical Research Council Cambridge Stem Cell Institute University of Cambridge, Cambridge, United Kingdom; ^7^Centro Nacional de Investigaciones Cardiovasculares, Madrid, Spain; ^8^Department of Oncology and Hematology, Institute of Hematology “L. and A. Seràgnoli”, University-Hospital S.Orsola-Malpighi, Bologna, Italy

**Keywords:** acute myeloid leukemia, bitter taste receptors, denatonium benzoate, bone marrow microenvironment, bitter compounds

In the original article, there was an error in the GEO database accession number. The correct GEO database accession number for gene expression data of denatonium-treated cells is GSE149548.

A correction has been made to the “Methods” section, paragraph 2, and the Data Availability Statement:

## Gene expression profiling (GEP)

TAS2R expression was analyzed in 61 AML, 49 from a published dataset (32) and 12 new cases. As validation set, we used also 183 AML samples downloaded from The Cancer Genome Atlas (TCGA) (https://gdc.cancer.gov/about-data/publications/laml_2012)(33). GEP after DEN treatment was performed in 5 newly diagnosed AML samples and THP-1 and OCI-AML3 cell lines. Three independent replicates of each condition were hybridized to Human Clariom S Arrays (Thermo Fisher Scientific) according to the manufacturer’s recommendations. Data quality control, normalization (signal space transformation robust multiple-array average), and supervised analysis were carried out by Expression Console and Transcriptome Analysis Console software, respectively (Thermo Fisher Scientific). For AML cells, data were normalized on vehicle-treated cells before comparison. Genes with a 1.5 fold difference and p ≤ 0.05 were considered for enrichment analyses. Downstream analyses were performed as reported in (32,34), and with Thomson Reuter’s MetaCore software suite (Clarivate Analytics, Philadelphia, PA, USA). Gene expression data of denatonium-treated cells will be publicly available on the GEO database under the accession number GSE149548.

## Data Availability Statement

The datasets presented in this study can be found in online repositories. The names of the repository/repositories and accession number(s) can be found below: the NCBI Gene Expression Omnibus (GSE149548).

The authors apologize for these errors and state that this do not change the scientific conclusions of the article in any way. The original article has been updated.

