# Supplementary material for: Denatonium as a Bitter Taste Receptor Agonist Modifies Transcriptomic Profile and Functions of Acute Myeloid Leukemia Cells
Source: Front Oncol. 2020 Jul 24;10:1225. doi: 10.3389/fonc.2020.01225 (PMC7393209; doi:10.3389/fonc.2020.01225)
Supplement: Supplementary file 1 [file Data_Sheet_1.docx]

**Figure S1**

**
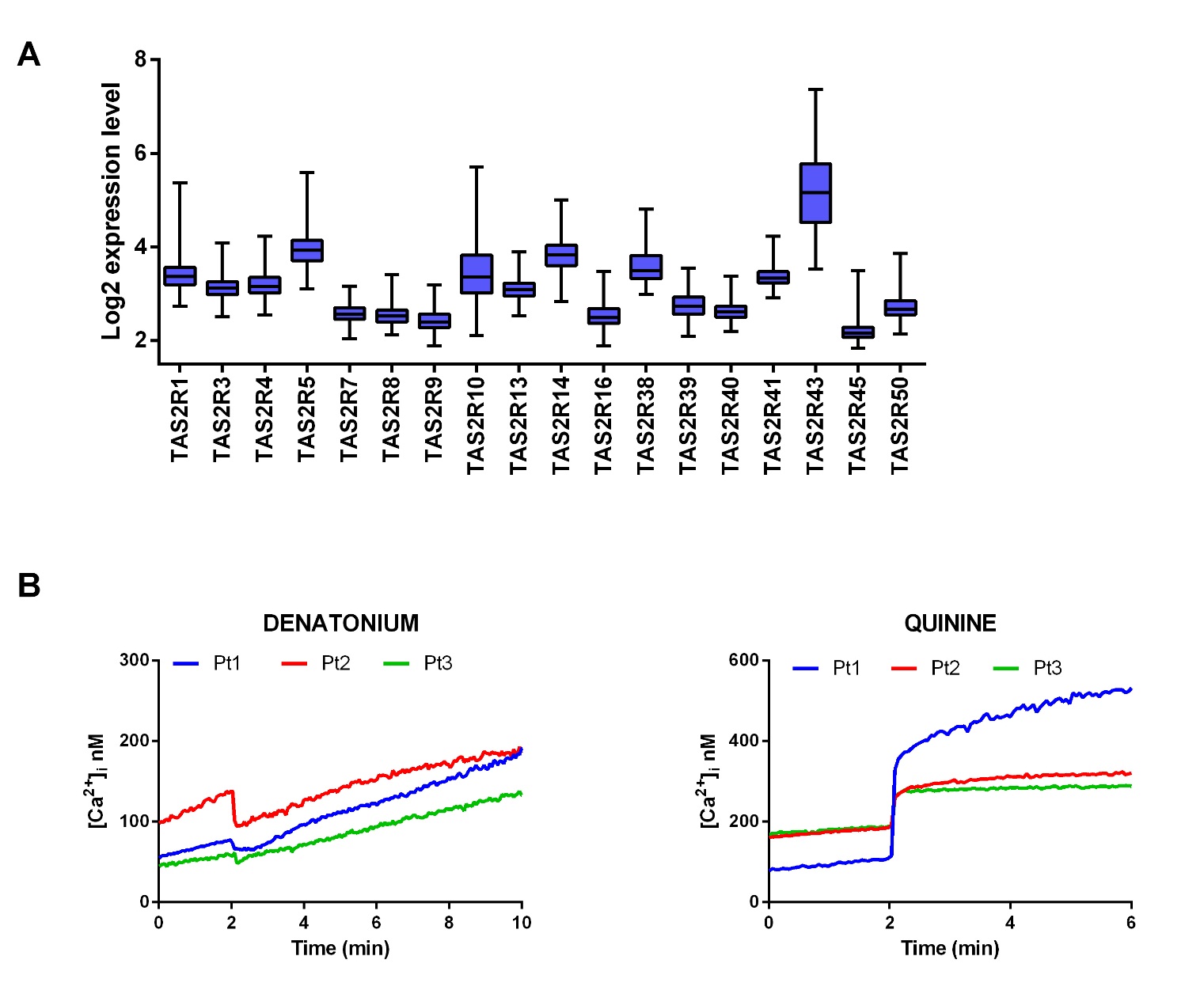
**

**Figure S1.** TAS2R expression in AML. **(A)** Box plots illustrate relative TAS2R mRNA expression obtained by GEP from the TCGA AML cohort consisting of 183 samples. (**B)** Ca^2+^ release in three AML samples (Pt) loaded with the Ca^2+^ indicator fura-2/AM and treated with 10mM denatonium or 75µM quinine.

**Figure S2**

**
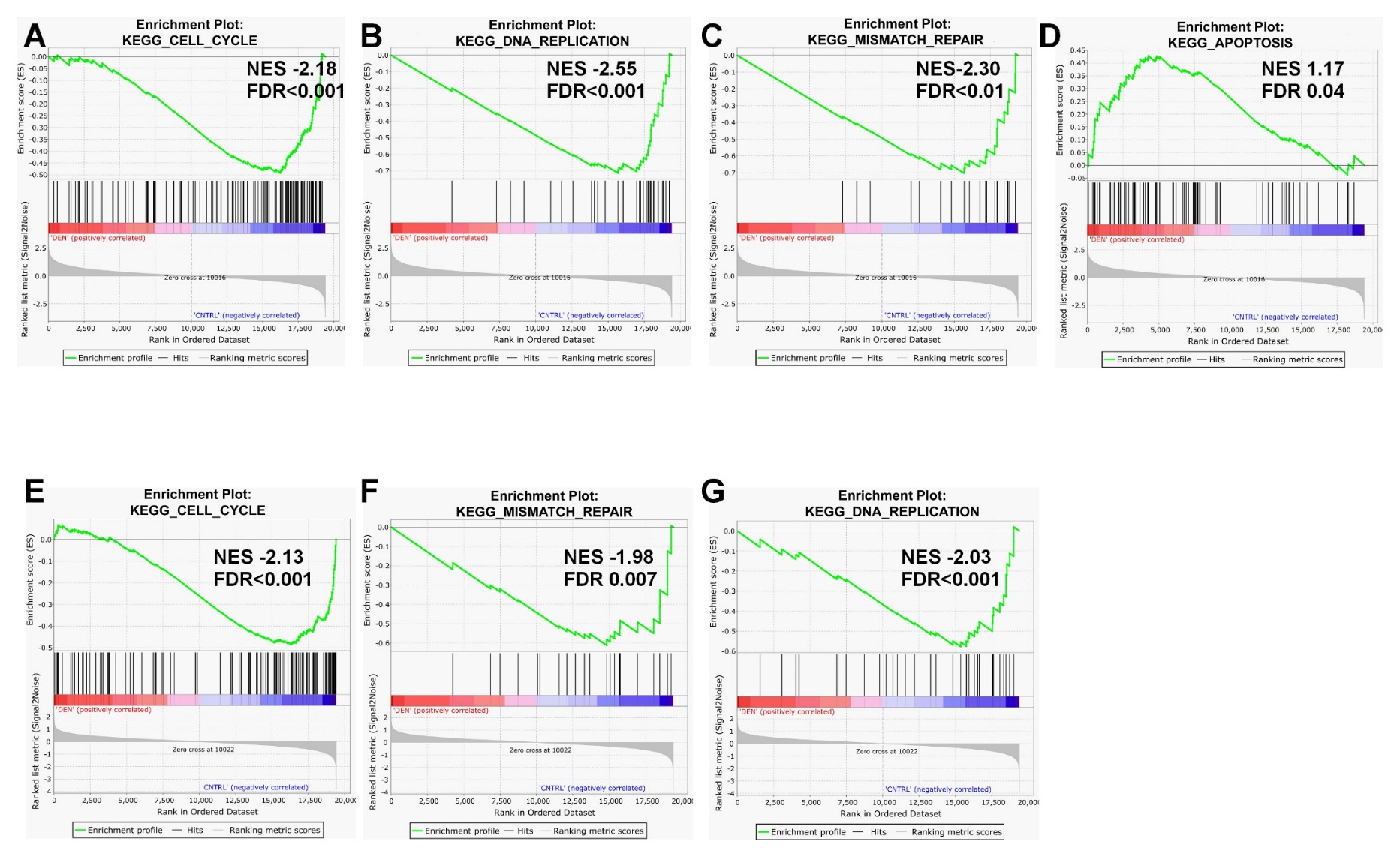
**

**Figure S2.** DEN exposure alters cell cycle, DNA replication, DNA repair, and apoptosis gene signatures. GSEA of DEN-treated and untreated THP1 **(A-D**) and OCI-AML3 cells **(E-G**) (signatures with significant false discovery rate enrichment are represented; NES: normalized enrichment score; FDR: false discovery rate).

**
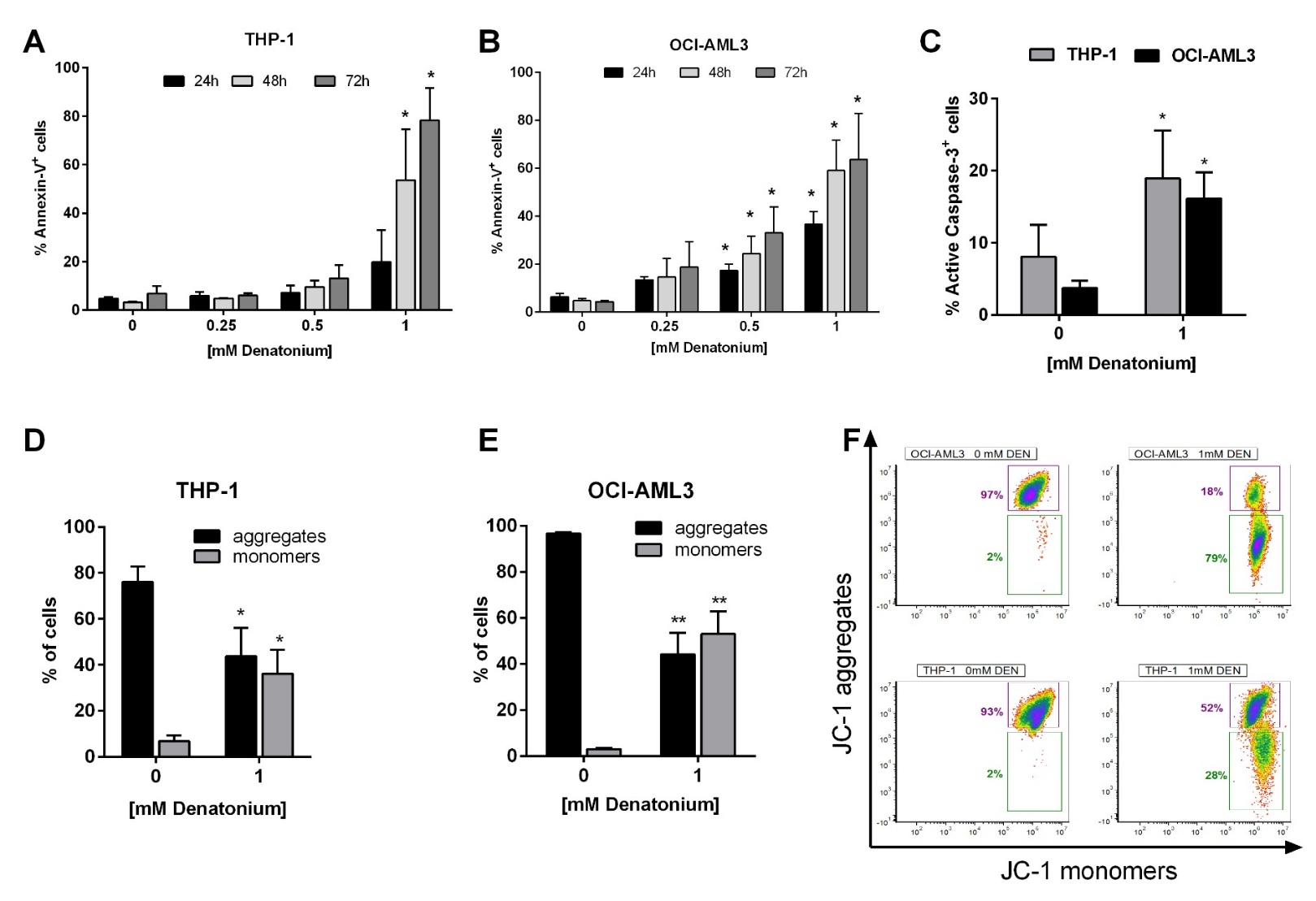
Figure S3**

**Figure S3.** DEN treatment induces AML cell line apoptosis. **(A)** THP-1 (n=4) and **(B)** OCI-AML3 (n=4) cell lines were treated for 24, 48 and 72 h with increasing doses of DEN. Annexin V/PI staining was used to detect apoptosis. **(C)** Active caspase-3 expression analyzed by flow cytometer after treatment with 1mM DEN for 48 h (n=5 for each cell lines). **(D-E)** Effect of treatment with 1mM DEN on transmembrane potential in mitochondria was detected by FACS analysis, in THP-1 (n=4) and OCI-AML3 (n=5) cell lines. The histograms show the percentage of JC-1 aggregates (cells emitting red fluorescence in the FL-2 channel) and JC-1 monomers (cells emitting green JC-1 detected in the FL-1 channel) from 6 independent experiments. **(F)** Representative dot plots of JC-1 staining. Data are expressed as mean ± SEM. Statistical analysis was performed by using ANOVA followed by Dunnett’s multiple comparison test with untreated group as control. *p<0.05; ** p<0.01.

**
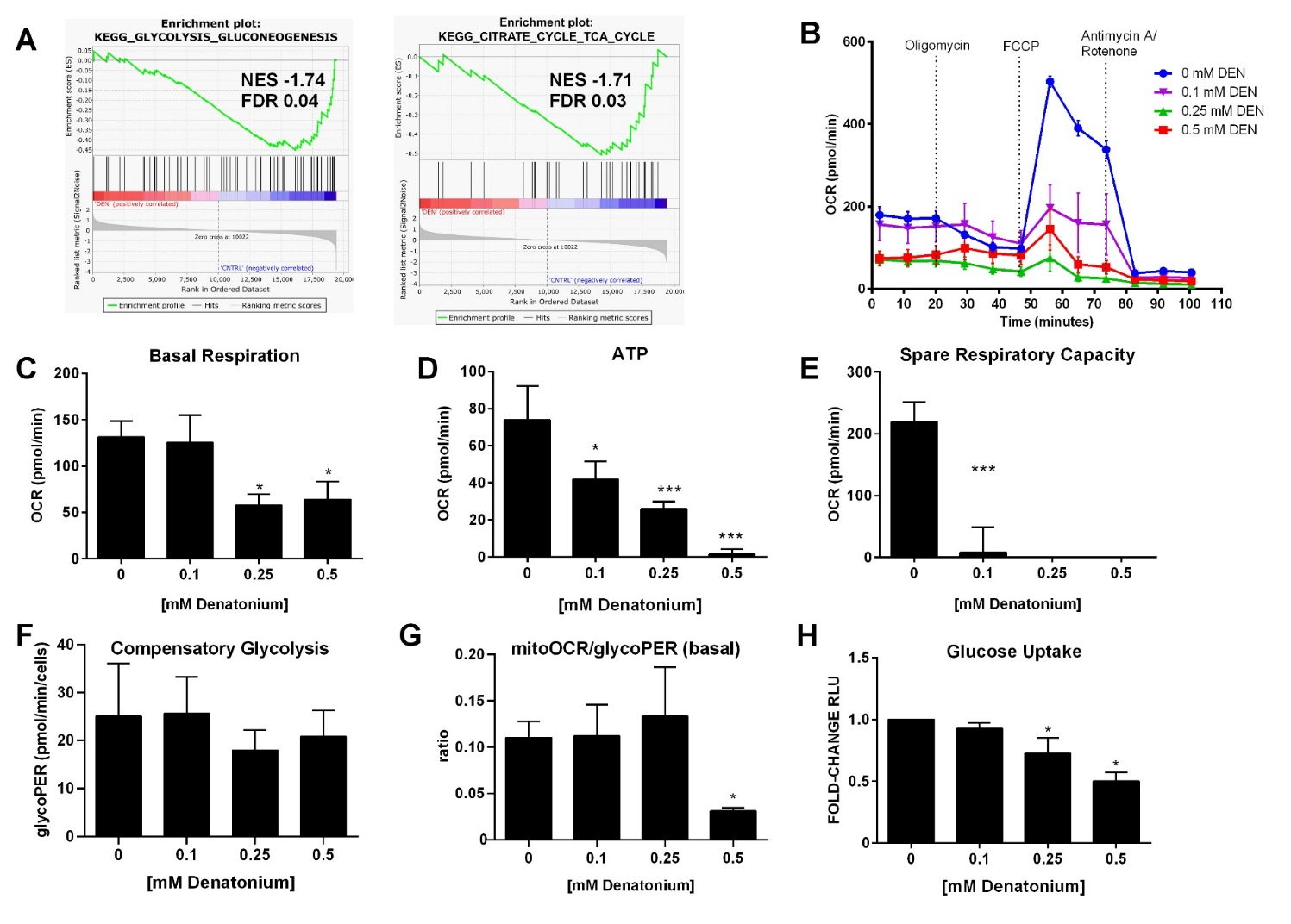
Figure S4**

**Figure S4.** DEN exposure alters mitochondrial bioenergetics. **(A)** Downregulation of a gene signatures of glycolysis/gluconeogenesis and citrate cycle in OCI-AML3 cells after 24 h exposure to DEN. **(B-E)** Seahorse Cell Mito Stress Test was performed to asses mitochondrial function in OCI-AML3 cell line (n=5) after 24 h exposure to increasing doses of DEN. **(B)** Representative oxygen consumption rate (OCR) profile plot. **(C)** Basal respiration. **(D)** ATP‐linked respiration. **(E)** Spare capacity. **(F-G)** Quantitative data of compensatory glycolysis and ratios of mitochondrial OCR to glycoPER calculated using Seahorse XF Glycolytic Rate assay (n=2). **(H)** Measurement of glucose uptake in OCI-AML3 cells after 24 h exposure to increasing doses of DEN (n=5). Untreated cells (136,730 ± 78,653 RLU) were used as reference and set as 1. Data are expressed as mean ± SEM. Statistical analysis was performed by using ANOVA followed by Dunnett’s multiple comparison test with untreated group as control. *p<0.05; ** p<0.01; ***p<0.001. NES: normalized enrichment score; FDR: false discovery rate.

**Figure S5**

**
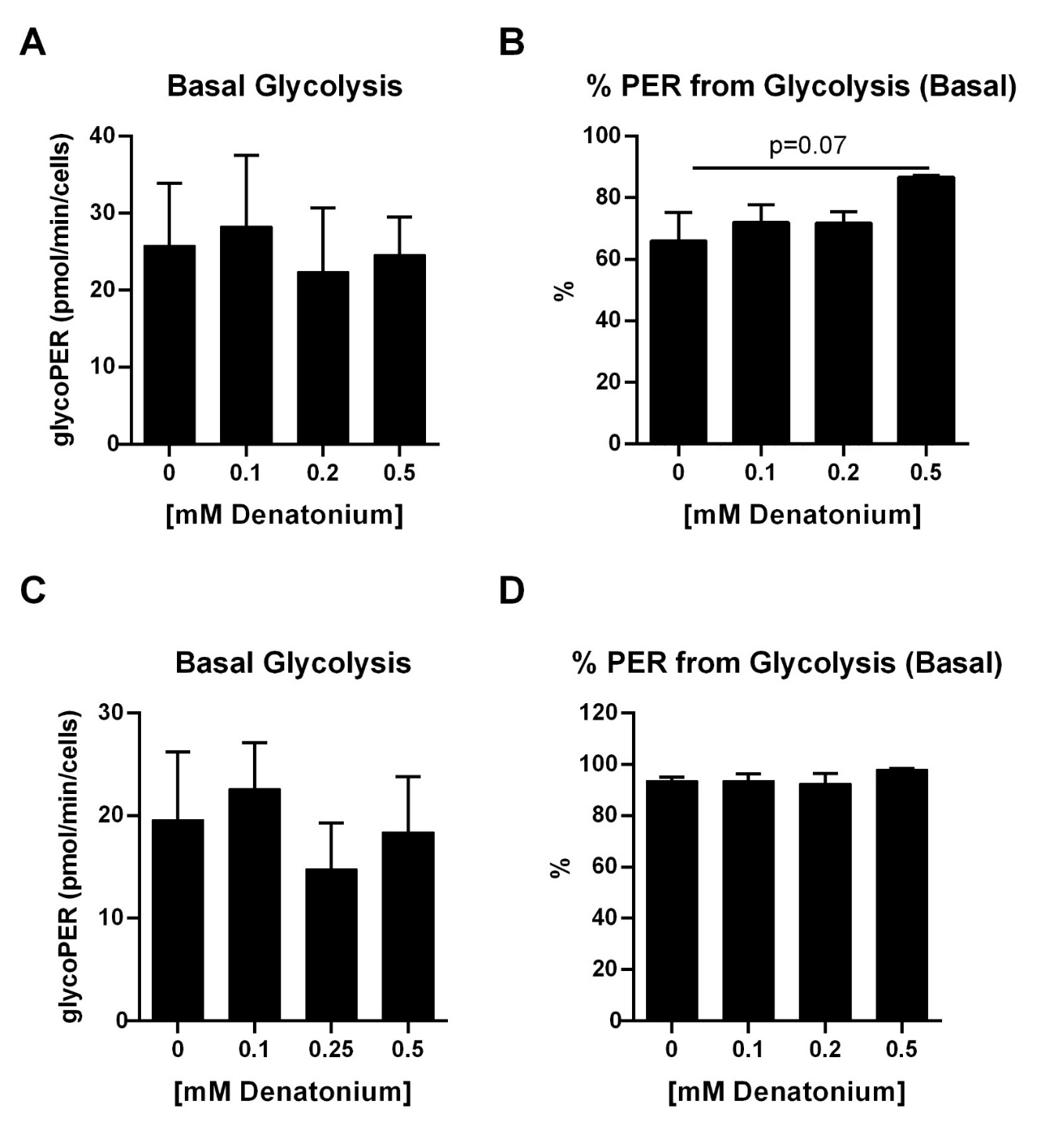
**

**Figure S5** Denatonium effects on glycolytic proton efflux rate (glycoPER) at basal. Individual parameters for basal glycolysis and basal percentage PER from glycolysis in THP1 cells (**A**-**B**, respectively; n=3) or in OCI-AML3 cells (**C-D**, respectively; n=2) treated with DEN at different concentrations. Data are expressed as mean ± SEM. Statistical analysis was performed by using ANOVA followed by Dunnett’s multiple comparison test with untreated group as control.

**Figure S6**

**
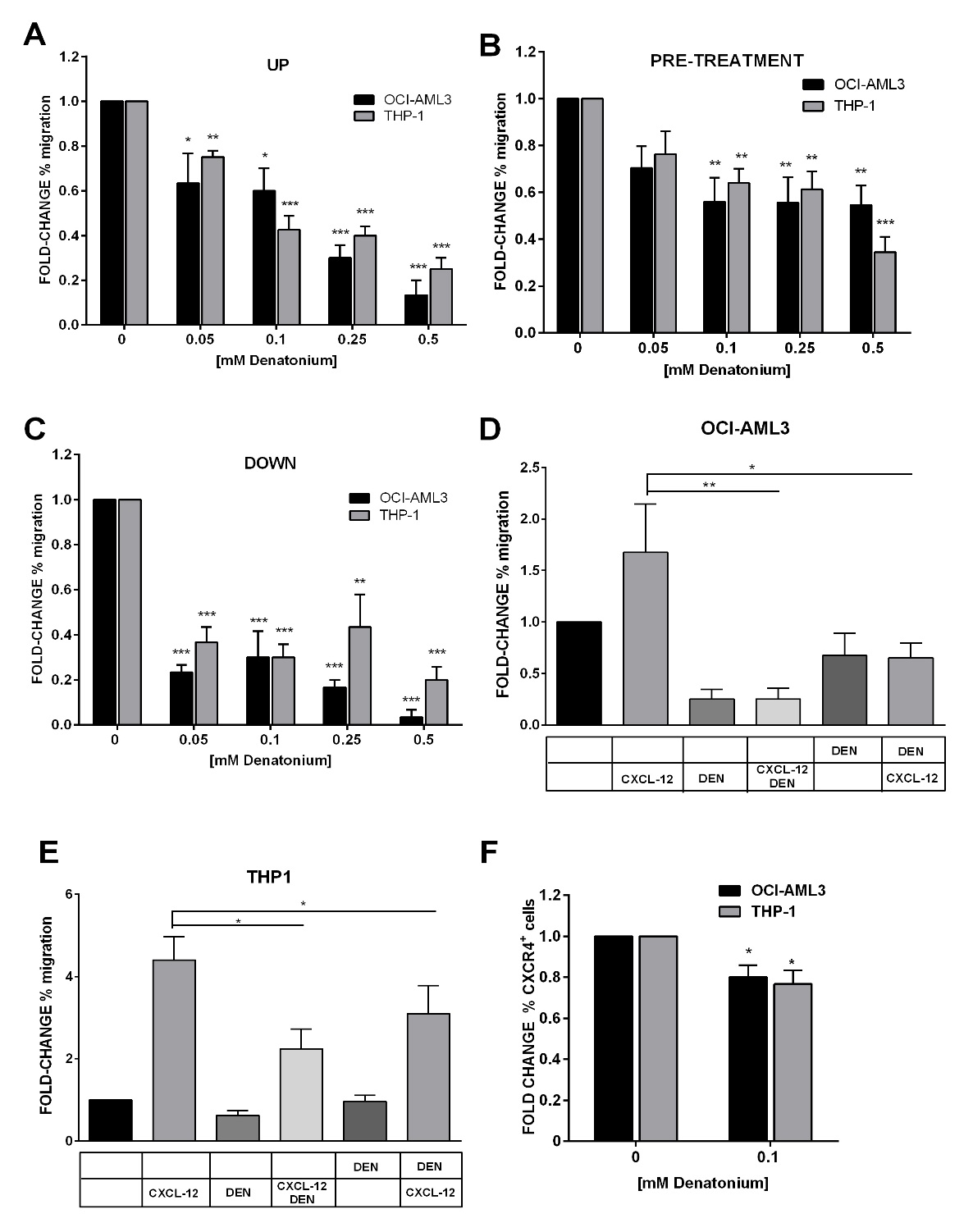
**

**Figure S6.** DEN stimulation inhibits OCI-AML3 and THP-1 cell lines migration. Results of cell lines migration in transwell assays were shown as fold-change of the percentage of migration in comparison with the untreated condition set as 1. **(A)** Spontaneous migration in presence of increasing doses of DEN in the upper chamber of transwell (n=4 for each cell lines). The percentage of migrating cells in control samples was: THP-1 10.6 ± 2.7 SEM and OCI-AML3 31.7 ± 8.7 SEM. **(B)** Spontaneous migration after a pre-treatment of 4 h with increasing doses of DEN (THP-1 n=3; OCI-AML3 n=4 ). The percentage of migrating cells in control samples was: THP-1 18.4 ± 2.8 and OCI-AML3 35.8 ± 8.6 SEM. **(C)** Migration towards a gradient of DEN in the lower chamber of transwell (n=3 for each cell lines). The percentage of migrating cells in control samples was: THP-1 10.6 ± 2.7 SEM and OCI-AML3 31.7 ± 8.7 SEM. **(D-E)** The histograms show the effect of the presence of 0.1mM DEN in the upper or in the lower chamber on the CXCL-12 induced chemotaxis (150ng/mL) (THP-1 n=8; OCI-AML3 n=4). The percentage of migrating cells in control samples was: THP-1 6.7 ± 2.9 SEM and OCI-AML3 32.8 ± 7.8 SEM. **(F)** CXCR4 expression analysed by flow cytometer after O/N treatment with DEN (THP-1 n=3; OCI-AML3 n=4). The percentage of CXCR4^+^ cells in untreated samples was: THP-1 61.5 ± 18.0 SD and OCI-AML3 77.6 ± 11.2 SD. Data are expressed as mean ± SEM. Statistical analysis was performed by using ANOVA followed by Dunnett’s multiple comparison test with untreated group as control. *p<0.05; ** p<0.01; ***p<0.001.
