# Supplementary material for: Denatonium as a Bitter Taste Receptor Agonist Modifies Transcriptomic Profile and Functions of Acute Myeloid Leukemia Cells
Source: Front Oncol. 2020 Jul 24;10:1225. doi: 10.3389/fonc.2020.01225 (PMC7393209; doi:10.3389/fonc.2020.01225)
Supplement: Supplementary file 2 [file Data_Sheet_2.docx]

**Table S1. Patient characteristics**

| **# PT** | **Cell source** | **Molecular abnormalities** | **Karyotype** | **WBC, cells/μL** | **Sex** | **Age** |
| --- | --- | --- | --- | --- | --- | --- |
| 1 | BM | NA | complex | 8.470 | F | 62 |
| 2 | PB | FLT3-ITD+, NPM1+, TP53 wt | NA | 64.250 | M | 70 |
| 3 | BM | FLT3-TKD+, NPM1+, DNMT3A+ (R882C) | +(4) | 129.000 | F | 60 |
| 4 | BM | FLT-3, NPM1, TP53 wt | t(6;14)(q25;q32), t(1;5)(p22;q33) | 1.540 | M | 67 |
| 5 | PB | FLT3-ITD+, FLT3 TKD wt, TP53 wt; IDH1 and IDH2 wt; DNMT3A+ (R882H), | normal | 68.780 | M | 69 |
| 6 | PB/BM | NPM1wt, FLT3 wt | +(8) | NA | M | 68 |
| 7 | BM | FLT3 wt, IDH1and IDH2 wt, NPM1 wt | t(6;14)(q25;q32), t(1;5)(p22;q33) | 2.300 | M | 38 |
| 8 | BM/PB | FLT3 wt, NPM1+ | normal | 25.300 | F | 63 |
| 9 | BM/PB | FLT3-ITD wt, FLT3-TDK wt, NPM1+ | NA | 1.600 | M | 59 |
| 10 | BM | FLT3 ITD, NPM1+ | normal | 90.800 | F | 73 |
| 11 | BM | FLT3-ITD wt, FLT3-TKD wt, IDH1+, IDH2 wt | normal | >30,000 | M | 19 |
| 12 | BM | PML-RARα | (15;17)(q22;q12), -Y | 8.060 | M | 43 |
| 13 | BM/PB | FLT3-ITD+ | normal | 70.000 | M | 65 |
| 14 | BM | NA | normal | NA | M | 79 |
| 15 | BM/PB | NA | normal | 260.000 | M | 72 |
| 16 | BM/PB | FLT-ITD+, NPM1+, WT-1 | normal | 46.000 | M | 31 |
| 17 | BM/PB | FLT3-ITD wt, FLT3-TKD+ , NPM1+, IDH1 wt, IDH2 wt, TP53 wt | normal | 118.780 | F | 61 |
| 18 | PB | NA | NA | 11.800 | M | 90 |
| 19 | BM | NA | NA | 33.000 | F | 74 |
| 20 | BM | NA | na | NA | F | 71 |
| 21 | BM | NA | normal | NA | F | 66 |
| 22 | BM/PB | FLT3 ITD wt, FLT3- TKD wt, NPM1+ | normal | 3.700 | F | 59 |
| 23 | PB | FLT3 wt, NPM1 wt | +(8) | 11.000 | M | 70 |
| 24 | BM/PB | NA | complex | NA | F | 72 |
| 25 | PB | NA | NA | 195.000 | M | 51 |
| 26 | BM | FLT3wt, NPM1wt e DNMT3A wt | +(19) | 12.500 | M | 72 |
| 27 | BM | FLT3-ITD | normal | 38.000 | F | 61 |
| 28 | BM | FLT3 wt, NPM1 wt | NA | 36.000 | M | 70 |
| 29 | PB | PML-RARα | t(15,17) | 99.300 | F | 77 |
| 30 | BM | FLT3-TKD+, NPM1 + | normal | 82.200 | F | 76 |
| 31 | BM | FLT3 wt, NPM1+, TP53 wt | normal | 1.700 | M | 73 |
| 32 | BM | FLT3-ITD+, NPM1+, TP53wt, IDH1 and IDH2wt | normal | 55.360 | M | 65 |
| 33 | BM | FLT3-TKD, NPM1+ | normal | 2.400 | F | 50 |
| 34 | BM | NA | iperdip | 2.440 | F | 74 |
| 35 | BM | NA | NA | 5.300 | F | 69 |
| 36 | BM | NA | complex | 238.000 | M | 69 |
| 37 | BM | FLT3 wt, NPM1 wt | del(7) | 1.400 | M | 61 |
| 38 | BM | FLT3-ITD, NPM1+ | normal | 95.000 | F | 50 |
| 39 | BM | FLT3 wt, NPM1 wt | normal | 88.000 | M | 45 |
| 40 | BM | FLT3 wt, NPM1 wt | normal | 37.200 | F | 38 |
| 41 | BM | FLT3 wt, NPM1 NA | complex | 7.360 | M | 61 |
| 42 | BM | FLT3 wt, NPM1 wt | complex | 71.810 | F | 62 |
| 43 | BM | FLT3 wt, NPM1 NA | normal | 660 | M | 64 |
| 44 | BM | FLT3-ITD, NPM1+ | normal | 189.500 | M | 63 |
| 45 | BM | NA | normal | 3.150 | F | 66 |
| 46 | BM | FLT3 wt, NPM1 na | normal | 4.000 | M | 74 |
| 47 | BM | FLT3 wt, NPM1 wt | normal | 3.800 | F | 51 |

**Legend**: PB, peripheral blood; BM, bone marrow; NA, Not available

**Table S2. RT-PCR probes used in this study**

| **Gene** | **accession number** | **purchased from** | **Probe ID** | **product size bp** |
| --- | --- | --- | --- | --- |
| TAS2R4 | NM_016944 | IDT | Hs.PT.58.24880205.g | 131 |
| TAS2R8 | NM_023918 | IDT | Hs.PT.58.27572746.g | 133 |
| TAS2R10 | NM_023921 | IDT | Hs.PT.58.25022966.g | 112 |
| TAS2R16 | NM_016945 | IDT | Hs.PT.58.3030778.g | 110 |
| TAS2R30/47 | NM_001097643 | IDT | Hs.PT.58.40967668.g | 147 |
| TAS2R38 | NM_176817 | IDT | Hs.PT.58.1997269.g | 122 |
| TAS2R39 | NM_176881 | IDT | Hs.PT.58.25428396.g | 123 |
| TAS2R43 | NM_176884 | IDT | Hs.PT.58.26835465.g | 137 |
| TAS2R46 | NM_176887 | IDT | Hs.PT.58.26491131.g | 142 |
| GAPDH | NM_002046 | IDT | Hs.PT.58.40035104 | 123 |
| TAS2R13 | NM_023920.2 | Applied Biosystem | Hs00256781_s1 | 132 |
| GNAT3 | NM_001102386.2 | Applied Biosystem | Hs01385397_m1 | 109 |
| GNB1 | NM_001282538.1 | Applied Biosystem | Hs00929799_m1 | 64 |
| PLC-b2 | NM_001284297.1 | Applied Biosystem | Hs00190117_m1 | 69 |
| GAPDH | NM_001289745.1 | Applied Biosystem | Hs00266705_g1 | 74 |

**Table S3. Primary and Secondary antibodies used in Western Blot Analysis**

| **Antibodies** | **Company** | **Catalog No.** | **Clone** | **Dilution** |
| --- | --- | --- | --- | --- |
| Goat anti-T2R4 | Santa Cruz Biotechnology | sc-169494 | polyclonal | 1:100 |
| Rabbit anti-T2R10 | Santa Cruz Biotechnology | sc-169473 | polyclonal | 1:100 |
| Goat anti-T2R47 | Santa Cruz Biotechnology | sc-34859 | polyclonal | 1:1000 |
| Mouse anti-PLC β2 | Santa Cruz Biotechnology | sc-515912 | B-2 | 1:500 |
| Goat anti-actin | Santa Cruz Biotechnology | sc-1616 | polyclonal | 1:5,000 |
| Rabbit anti-Phospho-Histone H2A.X | Cell Signaling Technology | 9718 | 20E3 | 1:1,000 |
| Rabbit anti-CDK2 | Cell Signaling Technology | 2546 | 78B2 | 1:2,000 |
| Rabbit anti-T2R8 antibody | Abcam | ab75109 | polyclonal | 1:400 |
| Rabbit anti-GNB1 | Abcam | ab137635 | polyclonal | 1:10,000 |
| Rabbit anti-T2R13 | ThermoFisher Scientific | PA5-39709 | polyclonal | 1:500 |
| Mouse anti-Cdc25A | ThermoFisher Scientific | MA5-13794 | DCS-120 | 1:200 |
| Mouse anti-Cyclin A2 | ThermoFisher Scientific | MA1-180 | 6B4D11 | 2µg/ml |
| Rabbit anti-Cyclin D1 | ThermoFisher Scientific | MA5-16356 | SP4 | 1:500 |
| HRP-conjugated donkey anti-goat IgG | Santa Cruz Biotechnology | sc-2033 | polyclonal | 1:50,000 |
| HRP-conjugated donkey anti-mouse IgG | Santa Cruz Biotechnology | sc-2314 | polyclonal | 1:50,000 |
| HRP-conjugated donkey anti-rabbit IgG | Bethyl | A120-108P | polyclonal | 1:50,000 |

| **Table S4. Core genes altered by Denatonium treatment at transcriptional level in both OCI-AML3 and THP-1 cells according to functional categories** | | |
| --- | --- | --- |
|  |  |  |
| **Pathway** | **Genes** |  |
| **Apoptosis** | TP53INP1, RNF130, CFLAR, TNFSF10, AIFM2 |  |
| **Cell cycle and DNA damage** | FRY, LIG4, AKAP9, ORC1, SPDL1, **CCNA2**, NCAPG2, RAD54B, NCAPG, KIF22, PBK, BTG3, CDC25A, CDC45, PLK1, CDC20, **CDC6**, CDCA3, **XRCC3**, CCNF, CCND1, CENPV, FANCG |  |
| **Cytoskeleton, cell adhesion and migration** | PIK3R5, TUBA8, FSCN1, HMMR, RHOF, EMD |  |
| **Metabolism** | DHCR24, ALDOC, MPI, MSMO1, GYS1, AKR1C2, INSIG1, BCKDK, **ACOT7**, SCD, FADS2, PNP, CPT2, LDHA, GPI, AKR1C1, PFKP, **RRM2**, FAM72A, MGAT4A, ST3GAL6, SPTLC3 |  |
| **Chromatin organization** | HIST1H2BB, HIST1H4D, HIST1H3I, HIST1H2BM |  |
| **Histone Methylation** | EZH2 |  |
| **Immune response** | TRIM22, DDX58, TLR4, RNASE6, TNFSF13B, GPR18, **C3** |  |
| **Signaling** | GPR3, ITPRIPL1, SDC4, MPZL1, RALGAPA2, DGKD, **GPR84** |  |
| **Transcription** | ETV4, ETV5, BARX1, ZNF394, SFMBT2, ZNF852, ZNF33B, ZNF546, |  |
| **Translation and post-translational modification** | FDFT1, PPME1, **DUSP5**, ATXN3, CTSS, RPS26, **DARS2** |  |
| **Protein degradation** | CLPP, HERC3, FAM63A |  |
| **Others** | NBEAL2, CPXM1, FAM83D, LDLR, SORL1, KCTD7, SLC1A3, FKBP4, NUP205, BORCS7, UCP2, VIT, CLDN15, ADGRL2 |  |
| **Bold genes** are deregulated also in primary AML samples by Denatonium treatment | |  |

| **Table S5. Pathways enrichment analysis for genes downregulated in primary AML cells after Denatonium exposure** | | |
| --- | --- | --- |
|  |  |  |
| **Pathway** | ***p* value** | **Genes** |
| **Cell cycle and DNA damage** | | |
| Cell cycle | 0,0090006 | CDC7, CCNE2, CDC6, MAD2L1, PCNA, PRKDC, CCNA2, MCM4 |
| G1/S transition of mitotic cell cycle | 5,18E-05 | CDC7, CCNE2, CDC6, TYMS, RRM2, PCNA, POLA1, USP37, POLA2, MCM4 |
| Regulation of transcription involved in G1/S transition of mitotic cell cycle | 5,22E-04 | CDC6, TYMS, RRM2, PCNA, POLA1 |
| DNA replication initiation | 1,73E-04 | CDC7, CCNE2, CDC6, POLA1, POLA2, MCM4 |
| DNA replication | 0,004543 | PCNA, POLA1, POLA2, MCM4, FEN1 |
| DNA strand elongation involved in DNA replication | 0,0017988 | GINS3, PCNA, POLA1, POLA2 |
| **Cytoskeleton, cell adhesion and migration** | | |
| Tight junction | 0,1182671 | IGSF5, CLDN18, MRAS, MYH6, JAM2, SRC |
| **Nucleotide biosynthesis** | | |
| Pyrimidine metabolism | 0,3154609 | TYMS, RRM2, POLA1, POLA2 |

| **Table S6. Pathways enrichment analysis for genes downregulated in OCI-AML3 cells after Denatonium exposure** | | |
| --- | --- | --- |
|  |  |  |
| **Pathway** | ***p* value** | **Genes** |
| **Cell cycle and DNA damage** | | |
| Cell cycle | 3,84E-10 | E2F1, CDC6, MAD1L1, PKMYT1, SMAD3, CDC20, PTTG1, CDC25C, CDC25A, WEE1, CDK2, CCNE2, CDC45, CCND1, RAD21, YWHAH, CDKN2A, PLK1, BUB1, BUB1B, ORC1, CCNA2 |
| Regulation of cell cycle | 8,88E-05 | MADD, FOXM1, PRR11, CCNF, PKMYT1, MYBL2, CDC25C, WEE1, CDC25A, JUNB, CCNE2, PLK1, CABLES2, CDK16 |
| Regulation of cyclin-dependent protein serine/threonine kinase activity | 9,87E-04 | CCNE2, CDC6, PKMYT1, CDC25C, CCNA2, SERTAD1, CDC25A |
| G1/S transition of mitotic cell cycle | 1,10E-05 | CDC6, PKMYT1, LATS1, CDK2, CDC25A, CCNE2, INHBA, MCM8, CCND1, CDC45, CDKN2A, RRM2, CDCA5, ORC1 |
| Regulation of transcription involved in G1/S transition of mitotic cell cycle | 0,00447953 | CDC6, CDC45, RRM2, KLF11, ORC1 |
| DNA replication initiation | 0,01472478 | CCNE2, CDC6, CDC45, GINS4, ORC1 |
| G2/M transition of mitotic cell cycle | 3,49E-06 | CEP72, FOXM1, TPX2, PKMYT1, CDC25C, LATS1, CDC25A, WEE1, CDK2, HMMR, PLK4, PLK1, TUBA4A, TUBG1, CIT, TUBA1A, MELK |
| Mitotic nuclear envelope disassembly | 0,00981002 | NDC1, PLK1, NUP205, BANF1, EMD, NUP58 |
| Sister chromatid cohesion | 1,93E-10 | MAD1L1, KIF22, DSN1, NUF2, KNTC1, SPDL1, CDC20, CENPE, AURKB, KIF2C, SPC25, CDCA8, RAD21, PLK1, INCENP, BUB1, BUB1B, SKA1, CLASP2, CDCA5 |
| Metaphase plate congression | 0,00480333 | FAM83D, KIF22, KIF2C, CENPE |
| **Cytoskeleton, cell adhesion and migration** | | |
| Microtubule-based process | 8,15E-05 | TUBA8, TUBB2A, TUBA4A, TUBA1A, TUBA1B, TUBA1C, TUBB3, GTSE1 |
| Cytoskeleton organization | 0,00337638 | CCL3, TUBB2A, NEDD9, TPM1, CCDC6, TUBA8, PAK4, TUBA4A, NCKIPSD, TUBA1A, TUBB3, TUBA1C, HIP1 |
| Gap junction | 0,00784101 | TUBA8, TUBB2A, TUBA4A, MAPK7, ITPR3, TUBA1A, TUBA1B, TUBA1C, TUBB3 |
| Microtubule-based movement | 0,01071636 | KIF23, KIF22, KIF2C, AP2A2, KIF4A, KIF15, KIF18B, CENPE |
| Positive regulation of Arp2/3 complex-mediated actin nucleation | 0,01701769 | WASF1, WASL, WAS |
| Adherens junction | 0,02802255 | TCF7, WASF1, ERBB2, SMAD3, WASL, SNAI1, WAS |
| **Metabolism** | | |
| Biosynthesis of unsaturated fatty acids | 0,03774614 | ACOT7, FADS1, SCD, FADS2 |
| Canonical glycolysis | 0,04169252 | GPI, ALDOC, ENO2, PFKP |

| **Table S7. Pathways enrichment analysis for genes downregulated in THP-1 cells after Denatonium exposure** | | |
| --- | --- | --- |
|  |  |  |
| **Pathway** | ***p* value** | **Genes** |
| **Cell cycle and DNA damage** | | |
| Cell cycle | 0,00363301 | KIFC1, XRCC3, DBF4, JAG2, PTTG2, ANKLE1, EIF4EBP1, ANK3, MLST8, CCNA2, ORC1, CDCA3, CIB1, GTPBP4, RAN, PLD6, TBRG4, PSMA2, MAD2L1, SPAG5, PSMA3, MAD2L2, MYBBP1A, TICRR, RCC1, PPAT, PSMB5, NCAPG2, PTK6, TDRKH, FBXO5, MNS1, NDRG1, EMD, TUBB4B, DIXDC1, GINS2, CDC20, SPDL1, SUV39H2, EIF4E, NOLC1, PSMC3, PLK1, SEC13, RAD54B, PARD6G, MYH10, KIF22, STOX1, EZH2, ZFP42, FAM83D, CCNE1, SPRY1, CDC45, PRMT1, PRMT5, RANBP1, ZPR1, FANCG, MYC, FOSL1, DHCR24, CDK1, CDC6, HSP90AA1, NUP88, CCNF, MEIOB, PBK, MCM2, UBE2C, FLNA, MCM6, CCND1, PSME2, RRM2, BTG4, NUP205, BTG3, TOP3A, SDCCAG3, RUVBL1, WDR43, UBE2S, BCAT1, CETN2, BCCIP, MLF1, HMMR, MIF, GPR3, RPA3, RPA2, NCAPG, FEN1, PINX1, TRIP13, MSH6, CDC25A, SLBP, CCNB1, PSMD14, MAPK12, SFPQ, CENPV, APBB2, RBM14, KIF20A |
| Regulation of cell cycle | 0,03969542 | STOX1, XRCC3, EZH2, FAM83D, CCNE1, PRMT1, CDC45, EIF4EBP1, PRMT5, RANBP1, MLST8, FOSL1, MYC, CCNA2, DHCR24, CDK1, CDC6, GTPBP4, CCNF, TBRG4, UBE2C, PSMA2, CCND1, MAD2L1, SPAG5, PSME2, JUN, BTG4, PSMA3, BTG3, SDCCAG3, MAD2L2, MYBBP1A, TICRR, KIAA0101, CETN2, BCCIP, BOP1, RCC1, MLF1, GPR3, MIF, RPA3, PSMB5, RPA2, PTK6, FBXO5, NDRG1, FEN1, HSPA8, CDC20, CDC25A, CCNB1, PSMD14, EIF4E, MAPK12, PLK1, PSMC3, SFPQ, CENPV, PES1, RBM14, APBB2 |
| G1/S transition of mitotic cell cycle | 0,0010724 | BCAT1, CDC6, CDK1, DBF4, EZH2, MCM2, RCC1, CDC25A, PPAT, RPA3, MCM6, CCNB1, CCNE1, RPA2, CDC45, PRMT1, EIF4EBP1, CCND1, EIF4E, RRM2, FBXO5, RANBP1, ZPR1, ORC1 |
| DNA replication | 0,01127578 | RPA2, RFC3, POLD2, MCM2, RNASEH2A, FEN1, MCM6, RPA3 |
| DNA damage response, detection of DNA damage | 0,03415096 | MRPS26, RPA2, RFC3, POLD2, DNAJA1, RPA3 |
| DNA repair | 9,47E-04 | POLR2H, KIF22, POLR2G, HIST1H4L, XRCC3, TICRR, UNG, MORF4L2, POLR2I, KIAA0101, CETN2, BCCIP, POLR2D, ANKLE1, STUB1, SMUG1, RPA3, RPA2, CDC45, HIST1H4A, HIST3H2A, FANCG, HIST1H4D, HIST3H3, FEN1, TRIP13, CIB1, CDK1, GINS2, SSRP1, MSH6, UFD1L, MEIOB, RNASEH2A, RFC3, PSMD14, SFPQ, POLD2, TOP3A, RAD54B, RUVBL2, RUVBL1, MAD2L2, RBM14, UBE2T |
| Spindle checkpoint | 0,01311789 | CCNB1, XRCC3, MAD2L1, PLK1, SPDL1, NDRG1, MAD2L2 |
| Metaphase/anaphase transition of cell cycle | 0,04007504 | CCNB1, CDC6, XRCC3, MAD2L1, PLK1, UBE2C, MAD2L2 |
| Chromosome segregation | 0,05588301 | KIFC1, KIF22, FAM96B, XRCC3, PTTG2, RCC1, ANKLE1, FAM83D, NCAPG, FEN1, PINX1, TRIP13, CDC6, MEIOB, CDC20, SPDL1, UBE2C, SRPK1, CCNB1, MAD2L1, PLK1, SPAG5, SFPQ, SEC13, MAD2L2 |
| Cell division | 0,11378695 | KIFC1, STOX1, CETN2, RCC1, FAM83D, CCNE1, ANK3, NCAPG, NCAPG2, FBXO5, CCNA2, MYC, ETV5, CDCA3, CIB1, CDK1, DIXDC1, CDC6, RAN, CCNF, SPDL1, CDC20, UBE2C, CDC25A, CCNB1, CCND1, MAD2L1, SPAG5, PLK1, CENPV, SDCCAG3, PARD6G, RUVBL1, MAD2L2, UBE2S, KIF20A, MYH10 |
| **Glucose metabolism** | | |
| Glucose metabolic process | 1,08E-04 | PDK1, SORD, PFKFB4, ALDOC, PFKP, HK2, PGAM1, DLAT, SDHAF3, GOT2, GPI, TPI1, GOT1, AKR1A1, PGAM4, SLC25A10, PGM1, ENO2, SLC25A1, PDHA1, PGK1, MYC, FABP5, ENO1 |
| Canonical glycolysis | 3,08E-06 | GPI, TPI1, PFKFB4, ALDOC, ENO2, PGAM1, HK2, PFKP, PGK1, ENO1 |
| Gluconeogenesis | 2,87E-04 | GOT2, GPI, TPI1, GOT1, SLC25A10, ALDOC, PGAM4, PGM1, ENO2, PGAM1, SLC25A1, PGK1, ENO1 |
| **Energy production** | | |
| Acetyl-CoA metabolic process | 7,09E-05 | DLST, MVD, DLD, FASN, ACLY, MVK, DLAT, PDHA1, ACAT1 |
| ADP metabolic process | 7,18E-07 | LDHA, PFKFB4, ALDOC, HK2, PGAM1, PFKP, AK4, DDIT4, GPI, GALK1, TPI1, PGAM4, PGM1, ENO2, PGK1, MYC, ENO1 |
| ATP metabolic process | 5,92E-13 | NDUFB3, TSPO, LDHA, UQCRC1, PFKFB4, ALDOC, CYC1, COX7B, PGAM1, HK2, NDUFAB1, STOML2, HSPA1A, ATP5G1, UQCRFS1, UQCRQ, ATP5G3, NDUFS6, GALK1, TPI1, UQCR11, FXN, PGAM4, ENO2, MYC, HSPA8, ENO1, NDUFA6, NUDT5, CYCS, PFKP, COX4I1, AK4, DDIT4, NDUFA11, PPIF, GPI, NDUFV1, NDUFV2, DLD, PGM1, ATP5A1, PGK1, SURF1 |
| Citrate cycle (TCA cycle) | 0,0162031 | DLST, DLD, IDH2, ACLY, DLAT, PDHA1, FH |
| Oxidative phosphorylation | 1,92E-06 | NDUFB3, UQCRC1, NDUFA6, COX7B, CYCS, CYC1, NDUFAB1, COX4I1, UQCRFS1, UQCRQ, NDUFA11, PPIF, NDUFS6, UQCR11, FXN, NDUFV1, NDUFV2, DLD, MYC, SURF1 |
| Cellular respiration | 8,66E-10 | NDUFB3, UQCRC1, COX7B, CYC1, NDUFAB1, BNIP3, UQCRFS1, UQCRQ, MTFR2, NDUFS6, IMMP2L, UQCR11, FXN, IDH2, PDHA1, MYC, ETFA, FH, DLST, NDUFA6, CYCS, COX4I1, TBRG4, DLAT, NDUFA11, TRAP1, NDUFV1, NDUFV2, DLD, ADSL, MYBBP1A, SURF1 |
| Electron transport chain | 1,16E-05 | NDUFB3, UQCRC1, NDUFA6, COX7B, CYCS, CYC1, NDUFAB1, COX4I1, UQCRFS1, UQCRQ, NDUFA11, NDUFS6, IMMP2L, UQCR11, NDUFV1, NDUFV2, DLD, MYBBP1A, ETFA |
| Mitochondrial electron transport, NADH to ubiquinone | 0,01712677 | NDUFB3, NDUFS6, NDUFA6, NDUFV1, NDUFV2, DLD, NDUFAB1, NDUFA11 |
| Mitochondrial electron transport, ubiquinol to cytochrome c | 6,55E-04 | UQCRC1, UQCR11, CYC1, CYCS, UQCRFS1, UQCRQ |
| Mitochondrial respiratory chain complex assembly | 1,69E-04 | NDUFB3, COA4, NDUFA6, NDUFAB1, BCS1L, SDHAF3, TIMM21, NDUFA11, IMMP2L, NDUFS6, NDUFV1, NDUFV2, FOXRED1, AARS2, SURF1 |
| Respiratory electron transport chain | 8,97E-06 | NDUFB3, UQCRC1, NDUFA6, COX7B, CYCS, CYC1, NDUFAB1, COX4I1, UQCRFS1, UQCRQ, NDUFA11, NDUFS6, IMMP2L, UQCR11, NDUFV1, NDUFV2, DLD, MYBBP1A, ETFA |
| **Nucleotide biosynthesis** | | |
| Nucleoside metabolic process | 5,14E-17 | TSPO, LDHA, UQCRC1, HMGCR, CYC1, NDUFAB1, PGAM1, STOML2, CAD, UQCRFS1, PNP, UQCRQ, NDUFS6, ACOT7, UQCR11, FXN, PGAM4, DLG3, MYC, IMPDH2, MPP2, NUDT5, CYCS, PFKP, COX4I1, DDIT4, NDUFA11, PDCL3, DNPH1, NME1, PGM1, DLD, ADSL, SURF1, PRPS1, NDUFB3, AHCY, PFKFB4, ALDOC, COX7B, HK2, CTPS1, HSPA1A, ATP5G1, HPRT1, ACAT1, ATP5G3, PPAT, GALK1, TPI1, ATIC, ENO2, DHODH, PAPSS2, HSPA8, ENO1, NDUFA6, AK4, APRT, PPIF, GPI, NDUFV1, NDUFV2, GAMT, ATP5A1, PGK1, FPGS |
| Nucleotide metabolic process | 6,68E-11 | ADCY1, TSPO, LDHA, UQCRC1, HMGCR, NT5C3B, CYC1, LHCGR, NDUFAB1, PGAM1, PRDX5, STOML2, CAD, UQCRFS1, PNP, UQCRQ, AFMID, NDUFS6, ACOT7, UQCR11, FXN, PGAM4, DLG3, PDHA1, MYC, IMPDH2, MPP2, NUDT5, CYCS, PFKP, COX4I1, GMPR, DDIT4, NDUFA11, DNPH1, NME1, RRM2, PGM1, DLD, ADSL, SURF1, PRPS1, NDUFB3, PFKFB4, ALDOC, UNG, COX7B, HK2, CTPS1, HSPA1A, ATP5G1, HPRT1, SMUG1, ACAT1, ATP5G3, PFAS, PPAT, GALK1, TPI1, ATIC, DHODH, ENO2, PAPSS2, HSPA8, ENO1, NDUFA6, AK4, APRT, GART, PPIF, GPI, NDUFV1, NDUFV2, ATP5A1, PGK1, PAICS |
| Purine metabolism | 8,52E-05 | POLR2H, POLR2G, ADCY1, NT5C3B, POLR2I, POLR2D, HPRT1, PNP, PPAT, PFAS, ATIC, PAPSS2, IMPDH2, POLR3G, POLR3H, NUDT5, POLR1B, AK4, GMPR, APRT, GART, NME1, RRM2, POLD2, PGM1, ADSL, PAICS, PRPS1 |
| **Amino acid metabolism** | | |
| Alpha-amino acid metabolic process | 1,89E-07 | BCAT1, PYCRL, AHCY, ALDH18A1, BCAT2, GLUD1, PRDX4, CTPS1, CAD, AGMAT, ACAT1, PPAT, PFAS, AFMID, GOT2, GOT1, PLOD1, P4HA1, GCSH, GPT2, DLST, HAL, GGH, GART, PYCR1, SLC25A32, DLD, GAMT, FPGS, CBS |
| Glutamine family amino acid metabolic process | 4,40E-05 | PYCRL, ALDH18A1, GLUD1, GGH, HAL, CTPS1, CAD, AGMAT, PPAT, PFAS, GOT2, PYCR1, GOT1, FPGS |
| **Lipid metabolism** | | |
| Lipid metabolic process | 0,01124091 | TSPO, SC5D, CYB5R2, PDP2, CPT2, LDLR, CRABP1, HMGCR, NDUFAB1, STOML2, LSS, ALG8, SDC4, FDFT1, NDUFS6, AKR1C2, ACOT7, STARD4, AGPAT5, NANS, INSIG1, SLC22A4, PDHA1, HADH, PCYT2, AKR1C1, PIGA, DHCR24, MT3, PLD6, CBR4, GAL, CECR5, PNPLA3, SDC1, SQLE, DLD, ORMDL2, MVK, PCCB, SLC27A2, HSD17B10, MVD, CYP51A1, HMGCS1, TRIB3, ECHS1, ACAT2, STUB1, PDSS1, ACAT1, HADHB, MIF, ISYNA1, CERS1, XBP1, DHCR7, APOC4, FASN, AGRN, AGK, NSDHL, ETFA, PDK1, EBP, LPL, MSMO1, FADS1, SCD, EEF1A2, MCAT, FDPS, FADS2, ACLY, DLAT, SOD1, OXSM, RDH11, AKR1B1, FABP3, HBEGF, LRP8, ATP5A1, IDI1, LIPE, FABP5, SMPD2 |
| Cholesterol metabolic process | 2,93E-07 | EBP, SC5D, MSMO1, LDLR, MVD, CYP51A1, HMGCR, HMGCS1, FDPS, LSS, ACLY, SOD1, FDFT1, SQLE, DHCR7, INSIG1, MVK, IDI1, LIPE, NSDHL, MT3, DHCR24 |
| Steroid metabolic process | 7,32E-05 | SC5D, TSPO, CYB5R2, MVD, LDLR, HMGCR, CYP51A1, HMGCS1, LSS, STUB1, FDFT1, AKR1C2, STARD4, DHCR7, INSIG1, AKR1C1, DHCR24, NSDHL, MT3, EBP, MSMO1, FDPS, ACLY, GAL, SOD1, SQLE, AKR1B1, MVK, IDI1, SLC27A2, LIPE |
| Fatty acid catabolic process | 0,03227633 | CPT2, ECHS1, ACAT2, HADH, ACAT1, SLC27A2, PCCB, LIPE, ETFA, HADHB |
